# Microwave-assisted synthesis of isosorbide-derived diols for the preparation of thermally stable thermoplastic polyurethane

**DOI:** 10.1080/15685551.2017.1395502

**Published:** 2017-11-13

**Authors:** Nejib Kasmi, Martina Roso, Nadia Hammami, Mustapha Majdoub, Carlo Boaretti, Paolo Sgarbossa, Chiara Vianello, Giuseppe Maschio, Michele Modesti, Alessandra Lorenzetti

**Affiliations:** ^a^ Laboratory of Interfaces and Advanced Materials (LIMA), Faculty of Sciences of Monastir – Boulevard of the Environment, University of Monastir, Monastir, Tunisia; ^b^ Department of Industrial Engineering & INSTM UdR Padova, University of Padova, Padova, Italy

**Keywords:** Isosorbide, sulphur, microwave assisted synthesis, polyurethane, thermal stability

## Abstract

In order to prepare thermally stable isosorbide-derived thermoplastic polyurethane, the synthesis of two new chiral exo–exo configured diols, prepared from isosorbide, and two types of diphenols (bisphenol A and thiodiphenol) was described. The synthesis conditions were optimized under conventional heating and microwave irradiations. To prove their suitability in polymerization, these monomers were successfully polymerized using 4,4′-diphenylmethane diisocyanate (MDI) and hexamethylene diisocyanate (HDI). Both monomers and polymers have been studied by NMR, FT-IR, TGA, DSC; intrinsic viscosity of polymers has also been determined. The results showed the effectiveness of the synthetic strategy proposed; moreover, a dramatic reduction of the reaction time and an important improvement of the monomers yield using microwave irradiation have been demonstrated. The monomers, as well as the polymers, showed excellent thermal stability both in air and nitrogen. It was also shown that the introduction of sulphur in the polyurethane backbone was effective in delaying the onset of degradation as well as the degradation rate.

## 1. Introduction

In the context of fossil-sourced monomers substitution, among the various biomass derived heterocyclic compounds, 1,4:3,6-dianhydrohexitols (DAHs) have a primary role [1–3]. Three isomers of the 1,4:3,6-dianhydrohexitol sugar diols exist, namely isomannide, isoidide and isosorbide. Isommanide exhibits *endo* and isoidide *exo* configuration for both the hydroxyl groups while isosorbide has one *exo* and one *endo* hydroxyl group, situated at carbons 2 and 5, respectively. Isosorbide is the sole compound among the three 1,4:3,6-dianhydrohexitols which is produced in industrial quantities, mainly because of the limited accessibility of the precursors of the other two isomers [1–3]. A rich variety of polyesters, polycarbonates, polyethers, polyamides and polyurethanes based on isosorbide has been described, recently complemented by epoxy resins [1–26]. The two hydroxyl functions of isosorbide show difference in reactivity [1,3]; for example, Javni et al. [17] showed that internal and hydrogen bonded *endo*-OH has lower reactivity toward phenyl isocyanate, based on steric consideration, while it has been proven that *endo* OH groups were more reactive in tosylation reaction [27]. This limits the exploitation of isosorbide as monomer in polymer synthesis because their difference in reactivity would often result in obtaining low molecular weight polymers and reduced crystallinity [11–21]. In order for isohexides to be successfully applied in commercial polymers, either new, milder polymerisation procedures or more reactive isohexide derivatives need to be developed [22]. According to Van Es [3], there are several strategies to overcome these drawbacks, with the aim of unlocking the full potential of isohexides in polymer chemistry. Among them, one strategy for increasing the reactivity of isohexides is the substitution of the relatively unreactive secondary hydroxyl groups at the 2- and 5-positions with more reactive ones. Hence, various difunctional isohexide derivatives were designed and reported in literature and most of them showed amine, carboxylic acid or isocyanate functionality [1,3–5,20–25]. In the same context, herein we describe a new strategy to synthesize two isoidide-based diols, prepared from isosorbide, having two hydroxyl functions in *exo*–*exo* position, therefore with the same reactivity and thus more suitable to be used in polymer synthesis. The effect of several aromatic comonomers (namely bisphenol A and thiodiphenol) has been investigated in order to improve both the hydroxyl reactivity and thermal stability of the isosorbide-derived diols. Using these modified diols, it is expected to obtain thermoplastic polyurethane with improved thermal stability. A similar approach has been reported very recently by Belgacem et al. for copolyethersulfones using bisphenol A as comonomer using conventional heating system [26]. Besides bisphenol A, we decided to use also thiodiphenol since very recently it has been found that incorporation of the sulfone moiety dramatically improves the thermal stability of the neat polyol [28]. Both conventional heating as well as microwave assisted synthesis have been considered.

## 2. Experimental

### 2.1. Materials and characterization

Isosorbide (98%, Acros Organics, France), 4-toluenesulfonyl chloride (98%, Sigma-Aldrich), potassium carbonate (99%, Acros Organics), bisphenol A (97%, Sigma-Aldrich), thiodiphenol (99%, Sigma Aldrich), sodium (Sigma Aldrich), pure 4,4′-methylenebis(phenyl isocyanate) (MDI), hexamethylene diisocyanate (HDI), anhydrous N,N-dimethylacetamide (DMAc), 99.8%, diethyl ether (99.8%) and dimethylformamide (DMF) (Acros Organics) were used as received. All the solvents used in this study were purchased from Sigma-Aldrich. ^1^H NMR and ^13^C NMR spectral data were obtained on a Bruker AV 300 spectrometer (Biospin). Fourier transform infrared (FTIR) spectra were acquired on a Nicolet iS50 FTIR (Thermo Fisher Scientific) system spectrometer by dispersing samples in KBr disks. Thermogravimetric analysis (TGA) was carried out on TA Instruments SDT Q600 at a heating rate of 10 °C·min^−1^ in nitrogen or air atmosphere; differential scanning calorimetry (DSC) analyses have been carried out on DSC Q200 (TA Instruments) using a 10 °C/min heating rate. Intrinsic viscosity [*η*] measurements of the polyurethane prepared were performed with an Ubbelohde viscometer at 25 °C in a mixture of phenol and tetrachloroethane (60/40 w/w). The samples were kept in the above mixture of solvents at 60 °C for 15 min to achieve complete dissolution; the solution was then cooled to ambient temperature and tested. For each sample, three different measurements were performed and the average value was calculated.

### 2.2. Synthesis

**Scheme 1.**
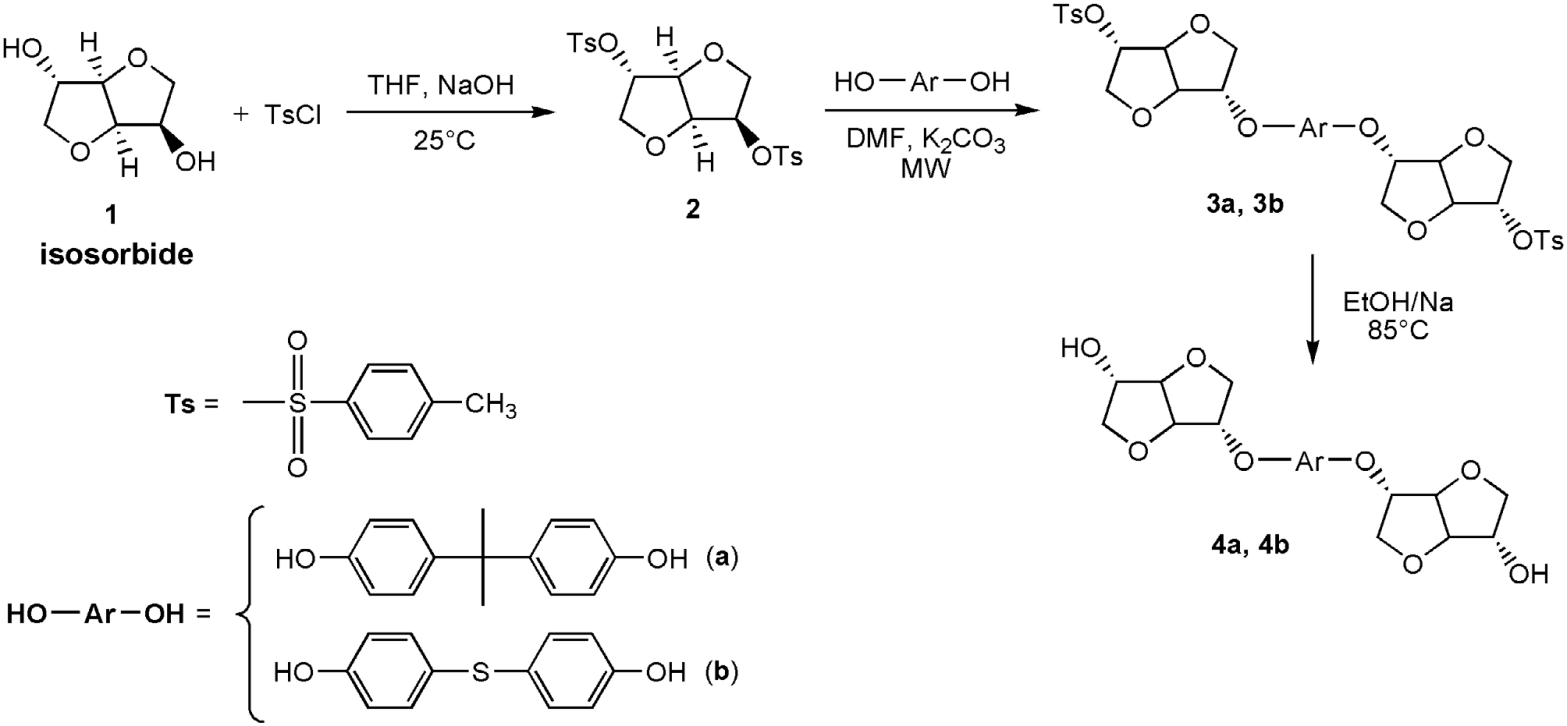
Synthetic route to the isosorbide based diols **4a** and **4b** (Δ and MW stay for conventional and microwave heating, respectively).

The synthetic pathway we followed to obtain the two modified diols (4a, 4b) is reported in Scheme 1.

#### 2.2.1. Synthesis of di-tosylated Isosorbide (2)

In a necked flask equipped with condenser and maintained under argon, 5 g of isosorbide (34 mmol) and 19.4 ml of an aqueous solution of NaOH (5 M) were charged. The mixture was kept under vigorous magnetic stirring in an ice bath, and then 70 mmol of 4-toluenesulfonyl chloride by small fraction dissolved in THF were added using a syringe. The solution was stirred for two hours at room temperature then evaporated; after that, the resulting mixture was recrystallized in water and dried at room temperature for 50 h under vacuum. The product was obtained as a white solid. Yield = 95%; M.p: 98 °C. calc. M (C_20_H_20_S_2_O_8_): 454.51 g.mol^−1^. ^1^H NMR (300 MHz, CDCl_3_, *δ*): 7.71 (m, 4H, H_g_, H_g’_), 7.28 (m, 4H, H_h_, H_h’_), 4.77 (m, 2H, H_b_, H_e_), 4.52 (m, 1H, H_c_), 4.41 (m, 1H, H_d_), 3.79–3.63 (3 m, 4H, H_a_, H_a′_, H_f_, H_f′_), 2.46 (s, 6H, H_i_); ^13^C NMR (75.5 MHz, CDCl_3_) *δ* (ppm): 145.51–145.37(C_12_,C_7_), 133.00–132.94(C_10_, C_15_), 130.14–129.98(C_8_, C_13_), 127.98–127.85(C_9_,C_14_), 85.59(C_2_), 83.26(C_5_), 80.3(C_4_), 78.31(C_3_), 73.25(C_6_), 69.78(C_1_), 21.71(C_11_).

#### 2.2.2. General procedure for the synthesis of the two intermediates (3a and 3b)

##### 2.2.2.1. Synthesis of intermediate (3a) by O-alkylation of bisphenol A (BPA) with ditosylated isosorbide (2)

In a 100 mL two-necked round bottom flask equipped with a condenser and an argon inlet, 5 mmol (1 eq) of bisphenol A (BPA), recrystallized from toluene (1.145 g), were added to 20 mL of DMF. The mixing has been carried out at room temperature under inert atmosphere (argon). After addition of the base K_2_CO_3_ (5 eq), 2.05 equivalents of ditosylated isosorbide **2** (4.65 g, 10.25 mmol) were added and the temperature was increased. Several isothermal reaction conditions, in the range 75–120 °C, have been investigated. For comparison purposes, this reaction, using the same reagents, has also been carried out under microwave heating using a multimode microwave reactor (Milestone Microsynth) and employing sealed stirred vessels. The temperature inside the vessels has been measured by using fiber optic sensor. The experiment has been carried out in temperature-control mode. The reaction time has been increased up to seven days under conventional heating while extremely shorter time (no more than 8 h) have been used for microwave heating. After the end of reaction, the mixture was precipitated in 150 ml of water under strong magnetic stirring. Then, several centrifugation steps have been carried out and the obtained product was dried and then dissolved in chloroform, dried with MgSO_4_ and concentrated. The desired product **3a** was obtained by precipitation in methanol, then filtered and washed with cold methanol. After drying in vacuum at 60 °C a white powder was obtained. As it will be shown in the following, the yield was up to 60% for conventional heating and up to 70% for microwave heating.

Calc. M (**3a**): C_41_H_44_O_12_S_2_ = 792.91 g mol^−1^. ^1^H NMR (300 MHz, CDCl_3_) *δ* (ppm): 7.85–7.81 (d, 4H, H_k_), 7.41–7.37 (d, 4H, H_j_), 7.17–7.13 (d, 4H, H_h_), 6.83–6.78 (d, 4H, H_g_), 4.97–4.95 (m, 2H, H_e_), 4.77–4.75 (m, 4H, H_b_ + H_c_), 4.69–4.67 (m, 2H, H_d_), 4.07–3.89 (m, 8H, H_a_ + H_f_), 2.49 (s, 6H, H_l_), 1.64 (s, 6H, H_i_). ^13^C NMR (75 MHz, CDCl_3_) *δ* (ppm): 154.59(C_7_), 145.32(C_14_), 144.00(C_17_), 130.3(C_10_), 130.07(C_16_), 127.94(C_15_), 127.86(C_9_), 114.68(C_8_), 85.42(C_2_), 85.36(C_5_), 82.94(C_4_), 80.69(C_3_), 72.62(C_6_), 72.15(C_1_), 41.76(C_11_), 30.94(C_12_), 21.67(C_13_).

##### 2.2.2.2. Synthesis of intermediate (3b) by O-alkylation of thiodiphenol with ditosylated isosorbide (2)

The synthesis and purification of **3b** has been carried out in the same way as **3a** using 1 eq of thiodiphenol instead of bisphenol A. As it will be shown in the following, the yield of **3b** was up to 58% for conventional heating and up to 72% for microwave heating.

Calc. M (**3b**): C_38_H_38_O_12_S_3_ : 782,15 g.mol^−1^. ^1^H NMR (300 MHz, CDCl_3_) *δ* (ppm): 7.85–7.81 (d, 4H, H_k_), 7.41–7.37 (d, 4H, H_j_), 7.31–7.27 (d, 4H, H_h_), 6.86–6.82 (d, 4H, H_g_), 4.97–4.95 (m, 2H, H_e_), 4.81–4.71 (m, 6H, H_b_ + H_c_ + H_d_), 4.07–3.91 (m, 2H, H_a_ + H_f_), 2.49 (s, 6H, H_i_). ^13^C NMR (75 MHz, CDCl_3_) *δ* (ppm): 156.14(C_7_), 145.44(C_12_), 132.91(C_15_), 130.15(C_13_), 128.48(C_14_), 127.91(C_10_), 127.22(C_9_), 116.04(C_8_), 85.32(C_2_), 82.86(C_5_), 82.74(C_4_), 80.84(C_3_), 72.51(C_6_), 72.21(C_1_), 21.75(C_11_).

#### 2.2.3. General procedure for the synthesis of the two diols (4a and 4b)

In a 250 ml flask fitted with a reflux condenser, 0.79 g (1 mmol) of **3a** was refluxed with 75 mL sodium ethoxide in ethanol (EtO^−^ Na ^+^, EtOH) (1 M) at 85 °C. The assembly was placed under argon. After 4 h, the detosylation of **3a** was completed. For purification, the ethanol was removed by rotary evaporation and the residue was dissolved, under magnetic stirring, in 100 mL of water and then it was treated with hydrochloric acid until acidic pH (4–5). The obtained product **4a** was filtered, under vacuum, and then it was recrystallized in toluene. The yield of **4a** was 65%.

Calc. M (**4a**): C_27_H_32_O_8_: 484.21 g.mol^−1^. M.p. = 132–134 °C. ^1^H NMR (300 MHz, CDCl_3_) *δ* (ppm): 7.12 (d, 4H, H_h_), 6.81 (d, 4H, H_g_), 4.82–4.80 (m, 4H, H_b_ + H_e_), 4.64–4.62 (m, 2H, H_c_), 4.41 (m, 2H, H_d_), 4.06–4.04 (m, 4H, H_a_), 3.94–3.93 (m, 4H, H_f_), 1.80 (s, 2H, OH), 1.66 (s, 6H, H_i_). ^13^C NMR (75 MHz, CDCl_3_) *δ* (ppm): 154.80(C_7_), 143.58(C_10_), 128.01(C_9_), 115.15(C_8_), 88.21(C_2_), 85.10(C_5_), 81.08(C_4_), 75.10(C_3_), 74.69(C_6_), 71.83(C_1_), 42.10(C_11_), 31.00(C_12_). FT-IR (KBr, cm^−1^): 3400 (w, OH stretching), 3050 (aromatic C–H stretching), 2960 and 2865 (w, aliphatic C–H stretching), 1598 and 1505 (s, C = C stretching), 1218 (s, C–O deformation vibration), 1057 (s, C–O–C asymmetric stretching), 811 (s, C–H out of plane bending). Microanalysis: (C_27_H_32_O_8_) Calc.: C, 66.91; H, 6.61; O, 26.48. Found: C, 66.05; H, 6.81; O, 26.55.

The synthetic pathway to prepare **4b** is the same as for **4a**. In this case, the reaction is completed after 3 h and the recrystallization has been carried out in diethyl ether. The yield was 62%.

M (**4b**): C_24_H_26_O_8_S = 474.13 g.mol^−1^. M.p. = 149–151 °C. ^1^H NMR (300 MHz, DMSO-d_6_) *δ* (ppm): 7.29 (d, 4H, H_h_), 6.92 (d, 4H, H_g_), 4.80–4.78 (m, 4H, H_b_ + H_e_), 4.64–4.62 (m, 2H, H_c_), 4.41 (m, 2H, H_d_), 4.06–3.93 (2 m, 8H, H_f_ + H_a_), 1.85 (s, 2H, OH). ^13^C NMR (75 MHz, DMSO-d_6_) *δ* (ppm): 156.39(C_7_), 132.89(C_9_), 128.28(C_10_), 116.15(C_8_), 87.94(C_2_), 85.02(C_5_), 81.39(C_4_), 76.00(C_3_), 74.60(C_6_), 72.27(C_1_). FT-IR (KBr, cm^−1^): 3431 (w, OH stretching), 3045 (aromatic C–H stretching), 2919, 2879 (w, aliphatic C–H stretching), 1598 and 1505 (s, C = C stretching), 1218 (s, C–O deformation vibration), 1070 (s, C–O–C asymmetric stretching), 829 (s, C–H out of plane bending). Microanalysis: (C_24_H_26_O_8_S) Calc.: C, 60.74; H, 5.50; O, 27.05; S, 6.71. Found: C, 60.19; H, 5.28; O, 27.44; S, 6.52.

#### 2.2.4. Procedure for the synthesis of the polyurethanes using diol (4a) and (4b)

In order to prove the suitability of the new diols to be used in preparing thermally stable polyurethane, the diol **4a** (1 mmol, 0.484 g) or **4b** (1 mmol, 0.474 g) and pure 4,4′-Methylenebis(phenyl isocyanate) (MDI) (1.05 mmol, 0.266 g) or HDI (1.05 mmol, 0.176 g) were dissolved in dry dimethylacetamide (10 mL). One drop of the catalyst dibutyltin dilaurate (SnDBDL) was added to this solution. The reaction mixture was flushed with nitrogen flow and heated to 80 °C for 24 h. After cooling down to room temperature, the reaction product was precipitated into cold methanol. The isolated polyurethane was dried at 25 °C under vacuum. The yield was 86 and 81% for MDI-based TPUs using **4a** and **4b**, respectively, and 79 and 83% for HDI-based ones using **4a** and **4b**, respectively. The polymers were named considering the isocyanate and the aromatic comonomer used; for example PU(MDI)BPA indicates a polyurethane obtained by reacting MDI with diol **4a**, i.e., obtained using BPA as aromatic comonomer.

**PU(MDI)BPA:** ^1^H NMR (300 MHz, DMSO-*d*_*6*_): *δ* (ppm): 1.24 (s, 6 H, H_i_), 3.69–5.28 (6 m, 18 H, isosorbide protons (H_a_-H_f_) and H_m_ of MDI), 6.47–6.88 (2 m, 8H, H_p_ and H_o_), 7.02–7.38 [4 m, 16 H, aromatic protons (H_g_,H_h_, H_k_ and H_l_)], 8.55 (s, urea proton H_n_), 9.76 (s, urethane proton H_j_). ^13^C NMR (75 MHz, DMSO-d_6_) *δ* (ppm): 29.5(C_12_), 31.13(C_18_), 41.71(C_11_), 71.86(C_1_), 74.72(C_6_), 75.11(C_3_), 81.21(C_4_), 85.14(C_5_), 88.18(C_2_), 114.47(C_8_), 118.79(C_16_), 128.11(C_23_), 129.04(C_20_), 129.39(C_15_), 129.53(C_9_), 135.42(C_22_), 136.23(C_17_), 137.9(C_14_), 138.13(C_21_), 143.63(C_10_), 147.07(C_19_), 153.03(C_13_), 154.98(C_7_). FT-IR (KBr, cm^−1^): 3304 (NH), 3029 (aromatic CH), 2921 (aliphatic CH), 1705 (C = O of urethane group), 1645 (C–N–H), 1595 (C = C–H), 1544, 1508 (C = C), 1233, 1177 (C–O–C).

**PU(HDI)BPA:** ^1^H NMR (300 MHz, DMSO-*d*_*6*_): *δ* (ppm): 1.25–1.32 (6 m, 16H, H_l_ and H_m_), 1.6 (s, 6H, H_i_), 2.95 (m, 8H, H_k_ and H_k’_), 3.70–5.25 (6 m, 16 H, H_a_–H_f_), 5.74 (urea proton H_n),_ 6.86–6.90 (m, 4H, H_g_), 7.12–7.17 (m, 4H, H_h_), 7.32 (urethane proton H_j_). ^13^C NMR (75 MHz, DMSO-d_6_) *δ* (ppm): 26.54(C_16_), 29.76(C_15_), 30.50(C_15′_), 31.13(C_12_), 40.83(C_14_), 41.25(C_14′_), 41.70(C_11_), 72.06(C_1_), 74.71(C_6_), 77.47(C_3_), 81.20(C_4_), 85.14(C_5_), 88.17(C_2_), 115.21(C_8_), 128.11(C_9_), 143.76(C_10_), 154.88(C_7_), 154.97(C_17_), 158.55(C_13_). FT-IR (KBr, cm^−1^): 3331 (–NH), 3036 (aromatic CH), 2934 (aliphatic CH), 1719 (C = O of urethane group), 1620 (C–N–H), 1577 (C = C–H), 1508 (C = C), 1248, 1183, 1075 (C–O–C).

**PU(MDI)THIO:** ^1^H NMR (300 MHz, DMSO-*d*_*6*_): *δ* (ppm): 3.69–5.26 (6 m, 18 H, isosorbide protons (H_a_–H_f_) and H_l_ of MDI), 6.47–6.88 (2 m, 8H, H_n_ and H_o_), 7.05–7.38 [16 H, aromatic protons (H_g_,H_h_, H_j_ and H_k_)], 8.55 (s, urea proton H_m_), 9.74 (s, urethane proton H_i_). ^13^C NMR (75 MHz, DMSO-d_6_) *δ* (ppm): 29.5(C_16_), 71.72(C_1_), 74.76(C_6_), 75.08(C_3_), 81.42(C_4_), 85.06(C_5_), 88.22(C_2_), 114.47(C_8_), 118.78(C_14_), 127.89(C_18_), 128.01(C_21_), 129.04(C_10_), 129.39(C_13_), 129.52(C_9_), 135.28(C_20_), 135.42(C_15_), 137.91(C_12_), 138.13(C_19_), 147.07(C_17_), 153.03(C_11_), 156.63(C_7_). FT-IR (KBr, cm^−1^): 3305 (NH), 3030 (aromatic CH), 2907 (aliphatic CH), 1705 (C = O of urethane group), 1644 (C–N–H), 1593 (C = C–H), 1545, 1508 (C = C), 1235, 1176, 1075 (C–O–C).

**PU(HDI)THIO:** ^1^H NMR (300 MHz, DMSO-*d*_*6*_): *δ* (ppm): 1.33 (m, 16H, H_k_ and H_l_), 2.95 (m, 8H, H_j_ and H_j′_), 3.36–5.30 (6 m, 16 H, isosorbide protons H_a_–H_f_), 5.73 (s, urea proton H_m),_ 6.98–7.01 (d, 4 H) and 7.27–7.31 (d, 4 H) aromatic protons (H_g_ and H_h_), 7.51 (s, urethane proton H_i_). ^13^C NMR (75 MHz, DMSO-d_6_) *δ* (ppm): 26.38(C_14_), 29.75(C_13_), 30.49(C_13′_), 42.38(C_12_), 43.47(C_12′_), 72.38(C_1_), 75.07(C_6_), 77.46(C_3_), 81.41(C_4_), 85.38(C_5_), 88.21(C_2_), 116.95(C_8_), 127.69(C_10_), 133.07(C_9_), 155.53(C_15_), 156.62(C_7_), 156.76(C_11_). FT-IR (KBr, cm^−1^): 3327 (–NH), 3062 (aromatic CH), 2934 (aliphatic CH), 1694 (C = O of urethane group), 1621 (C–N–H), 1572 (C = C–H), 1490 (C = C), 1239, 1180, 1076 (C–O–C).

## 3. Results and discussion

The strategy used here (Scheme 1) consists of an indirect transformation in order to have two identical hydroxyl functions in terms of the spatial configuration (i.e., both *exo*–*exo*) and therefore having similar reactivity. The monomers, which were soluble in most of organic solvents, were obtained by linking two L-idide moieties through two different aromatic rigid bridges, namely bisphenol A (BPA) and thiodiphenol (THIO). The chiral isosorbide-derived diols were synthesized following the three-step route described in Scheme 1. The tosylation of isosorbide to obtain compound **2** was performed under the conditions already reported in the literature [8,29], using 4-toluenesulfonyl chloride in aqueous NaOH/THF system. The structure of the obtained compound **2**, accordingly to what already reported in literature [8,27,29], was confirmed by ^1^H NMR and ^13^C NMR analyses (Figures S1 and S2 in the supplementary information). Two different diphenols (bisphenol A, BPA, and thiodiphenol, THIO) were then di-O-alkylated with tosylated isosorbide **2** in the presence of potassium bicarbonate as base and DMF as solvent. In view of the steric hindrance effect, the *exo* position reactivity was limited [30]. Consequently, a selective *endo*-monoalkylated isosorbide-containing derivatives **3a** and **3b** was obtained. In fact, compounds **3a** and **3b** are formed by an O-alkylation involving a nucleophilic substitution reaction of the SN2 type, which leads to inversion of configuration of the C-5 carbon atom (called Walden inversion); whereas the access to the rear of C-2, bearing an *exo*-tosylate group, is hindered by the isosorbide ring system, as already reported for similar reactions [30]. The difunctionalized derivatives having *exo*–*exo* diols configuration, **4a** and **4b,** were achieved by deprotection of compounds **3a** and **3b** using sodium ethoxide in ethanol [27], which is a protic polar solvent with strong ionizing and high resolving powers. The obtained monomers **4a** and **4b** showed good solubility in common organic solvents such as chloroform, acetone, methanol, ethanol and dichloromethane. All steps gave good yields in relatively simple reaction conditions, except the O-alkylation of BPA and THIO by the ditosylated isosorbide **2**, which required a reaction time up to seven days to reach yield between 55 and 60% when using the conventional heating. For this reason, this reaction has been further studied under microwave heating.

### 3.1. Optimization of the O-alkylation reaction conditions under conventional heating and microwave irradiations

#### 3.1.1. Study of the O-alkylation step under conventional heating: synthesis of intermediates (**3a, 3b**)

In order to optimize the synthesis conditions to obtain compounds **3a** and **3b** via the O-alkylation reaction, and thus to reduce reaction time and/or improve yield, the effect of the base excess, temperature and reaction time have been investigated.

##### 3.1.1.1. Base effect

According to what reported in literature [31], the O-alkylation reaction was carried out in DMF using potassium carbonate as a base. A stoichiometric ditosylated isosorbide (**2**)**/**diphenols mixture was reacted at 75 °C for 96 h in the presence of K_2_CO_3_. The effect of base concentration was studied using different excess of K_2_CO_3_. The results showed that increasing the base concentration the yield in products **3a** or **3b** was improved; however, no further effect was observed above 5 equivalents of base (Table 1). During these trials, thin-layer chromatography (TLC) indicated the presence of a spot aside from the known compounds in the reaction medium (diphenol, ditosylated isosorbide, di-O-alkylated diphenol and mono-O-alkylated diphenol) suggesting the presence of a side product due to elimination reaction. Indeed, even if tosylate isosorbide is less prone to elimination reaction than halide in case of alkylation [32], the formation of elimination products has already been reported in literature for similar synthesis [8,33].

**Table 1.** Effect of the amount of base (K_2_CO_3_) on the synthesis of **3a, 3b** (Stoichiometric **2/**diphenols mixture, 75 °C, 96 h).

Base amount/diphenols		3 eq	4 eq	5 eq	6 eq	7 eq
Yield (%)	**3a**	38	41	48	50	49
	**3b**	28	31	42	44	43

##### 3.1.1.2. Reaction temperature effect

Several O-alkylation reactions of the two diphenols with ditosylated isosorbide **2** at differents temperatures, in the K_2_CO_3_/DMF system, were carried out. The results are summarized in the Table 2 and showed a systematic decrease in the reaction yield when rising the temperature. Besides, the thin layer chromatography (TLC) analyses of reaction mixture indicated the formation of a side product, probably by elimination reaction, at temperature exceeding 75 °C. Indeed, such reactions are generally favoured when increasing temperature. We decided, therefore, to work under milder conditions.

**Table 2.** Effect of temperature on the synthesis of **3a, 3b** (Stoichiometric **2/**diphenols mixture, K_2_CO_3_/diphenols molar ratio: 5, 48 h).

Temperature (°C)		75	85	95	120
Yield (%)	**3a**	32	28	24	12
	**3b**	27	25	19	11

##### 3.1.1.3. Reaction time effect

In view of the results just reported, the effect of reaction time has been studied for O-alkylation reaction at 75 °C with 5 eq of K_2_CO_3_. The results in terms of yield are summarized in Table 3. It has been noticed that the longer the reaction time the higher the yield. The mono-O-alkylated diphenols by ditosylated isosorbide has been detected in the reaction mixture, after 7 days. A maximum of 60 and 58% reaction yields were achieved with **3a** and **3b**, respectively. To summarize, under conventional heating, reaction yields of **3a, 3b** no higher than 60% can be obtained even when using optimized conditions (75 °C, 5 eq of K_2_CO_3_ per eq of diphenols (BPA or THIO) and time of reaction of 7 days). Moreover, also the formation of some byproducts was revealed.

**Table 3.** Effect of reaction time on the synthesis of **3a, 3b** (Stoichiometric **2/**diphenols mixture, K_2_CO_3_/diphenols molar ratio: 5, 75 °C).

Time (day)		1	2	3	4	5	7
Yield (%)	**3a**	29	32	36	48	52	60
	**3b**	26	27	29	42	47	58

#### 3.1.2. Optimization of O-alkylation reaction for synthesis of the two intermediates (3a, 3b) under microwave irradiations

In order to improve the yield, possibly avoiding the formation of byproducts and reducing the reaction time, the synthesis of the compounds **3a, 3b** was performed under microwave (MW) irradiation. Indeed, it is well known since many years that microwave heating can ‘accelerate’ organic chemical transformations, as well as improving yield and selectivity [34]. In particular, it has already been reported for systems similar to those we are examinating here that under MW heating the yield was considerably improved and the reaction time dramatically decreased as compared to traditional heating methods [32,35,36]. A systematic study was carried out with varying different parameters (base concentration, temperature and reaction time) under MW activation. Tables 4 and 5 summarize the results obtained. It is very important to note that the reaction time to obtain good yield under microwave heating was dramatically lower than under conventional heating: similar yield can be obtained in several hours instead of one week of reaction. The same conclusion has been reported in literature for alkylation reaction of similar system to obtain ethers and esters [32,35,36]. Accordingly, this effect should be attributed to an increase in system polarity from the ground state of the reaction to the transition state where ion pairing is much more loose. It results in an enhancement in polarity during the reaction progress and, consequently, to an increase of MW materials interactions magnitude responsible for the observed acceleration [37]. An improvement in the reaction yield of the synthesis of **3a** was observed with increasing both the reaction time and the temperature. The best condition to carry out the synthesis were reaction temperature of 95 °C and reaction time of 8 h. For all the conditions tested, no by-products were revaled except when exceeding 8 h at 95 °C. For this reason no higher temperature nor longer reaction time were investigated. The same general procedure reported for synthesis of **3a** under microwave irradiations has been applied to the O-alkylation reaction of **3b**. Based on the results obtained before for **3a**, in order to try to improve the yield, some O-alkylation reactions of thiodiphenol with **2** under MW heating have been carried out by changing temperature and time as shown in the Table 5. The product **3b** can be obtained with 72% as best yield. As before, side products were not detected in the reaction mixture using TLC until exceeding 8 h at 100 °C and for this reason no higher temperature nor longer reaction time were investigated. The expected structures of both **3a** and **3b** were confirmed by ^1^H NMR and ^13^C NMR analysis as shown in the Figures S3, S4, S5 and S6 (Supplementary Information).

**Table 4.** Reaction yields of the synthesis of **3a** under MW (Stoichiometric **2/**BPA mixture, K_2_CO_3_/BPA molar ratio = 5).

Temperature (°C)	75			85			95		
Reaction time (h)	4	6	8	4	6	8	4	6	8
Yield (%)	27	40	57	31	44	62	35	49	70

**Table 5.** Obtained reaction yields of the synthesis of **3b** under MW (Stoichiometric **2/**THIO mixture, K_2_CO_3_/THIO molar ratio = 5).

Temperature (°C)	85		95		100	
Reaction time (h)	7.5	8	7.5	8	7.5	8
Yield (%)	58	63	64	72	60	68

#### 3.1.3. Synthesis of isosorbide based monomers (4a, 4b)

The two difunctionalized diols **4a, 4b** having *exo*–*exo* configurations were obtained by deprotection of compounds **3a, 3b** using sodium ethoxide in boiling ethanol. Indeed, the inversion of configuration of the **C-5** bearing the *endo*-tosyl group (in compounds **3a** and **3b**) reported before results in the formation of an *exo* (C–O) bond in compounds **4a** and **4b** since only an *exo*-linkage can bridge the **C-5** of 1,4:3,6-Dianhydrohexitol unit with the adjacent oxygen atom. This is confirmed by the analysis of the number of signals observed in the ^1^H and ^13^C NMR spectra of diols **4a** and **4b** (see below) and by comparison with similar isoidide based compounds as reported in literature [7,38,39].

### 3.2. Characterization of the three isosorbide based diols

The chemical structures of the monomers **4a** and **4b** were confirmed by ^1^H, ^13^C NMR and FT-IR spectroscopic analyses and their thermal properties were investigated by TGA and DSC.

#### 3.2.1. ^1^H and ^13^C NMR characterization

**Figure 1.**
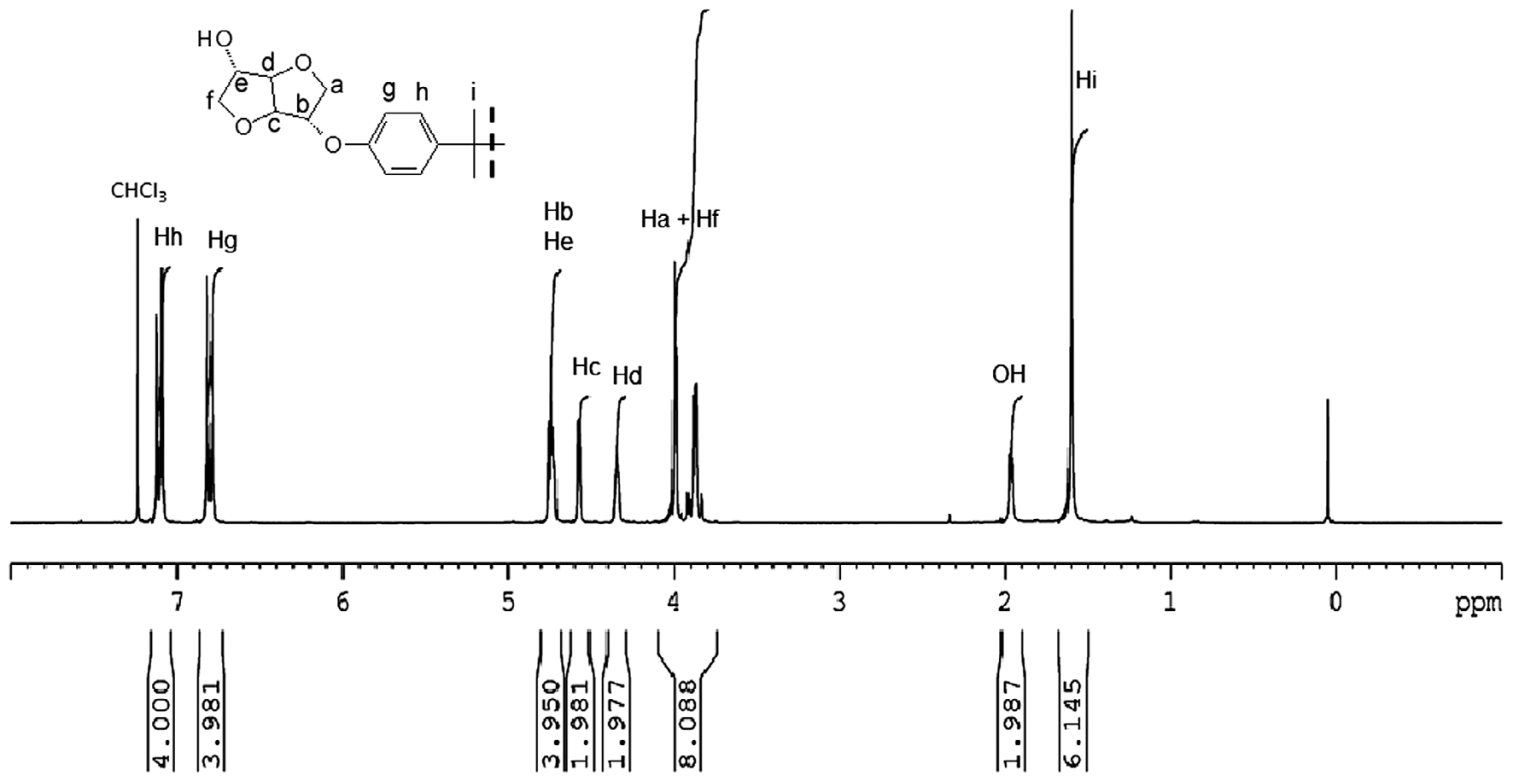
1H NMR spectrum of the monomer 4a (300 MHz, 293 K, CDCl3).

**Figure 2.**
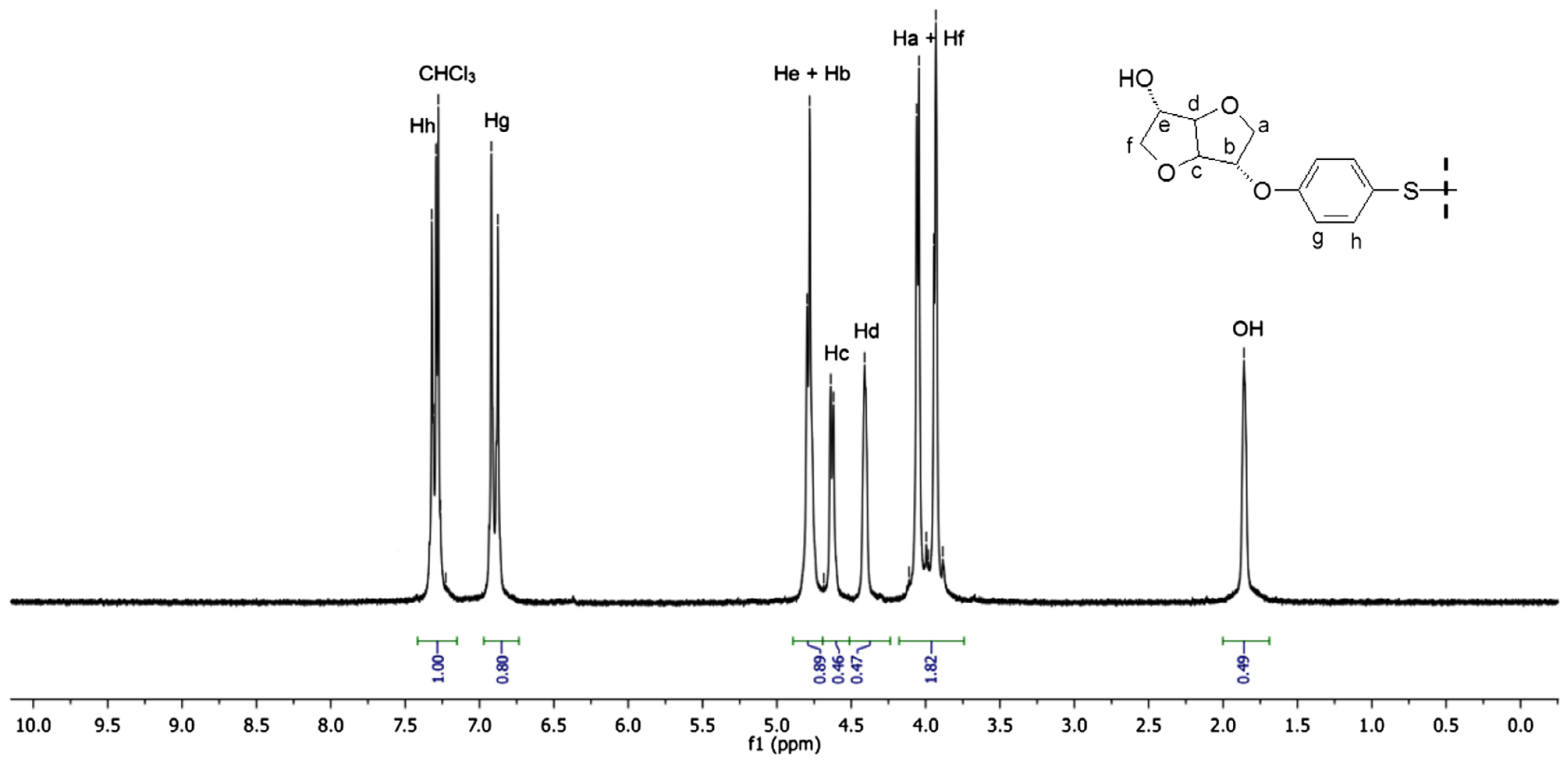
^1^H NMR spectrum of the monomer **4b** (300 MHz, 293 K, CDCl_3_).

**Figure 3.**
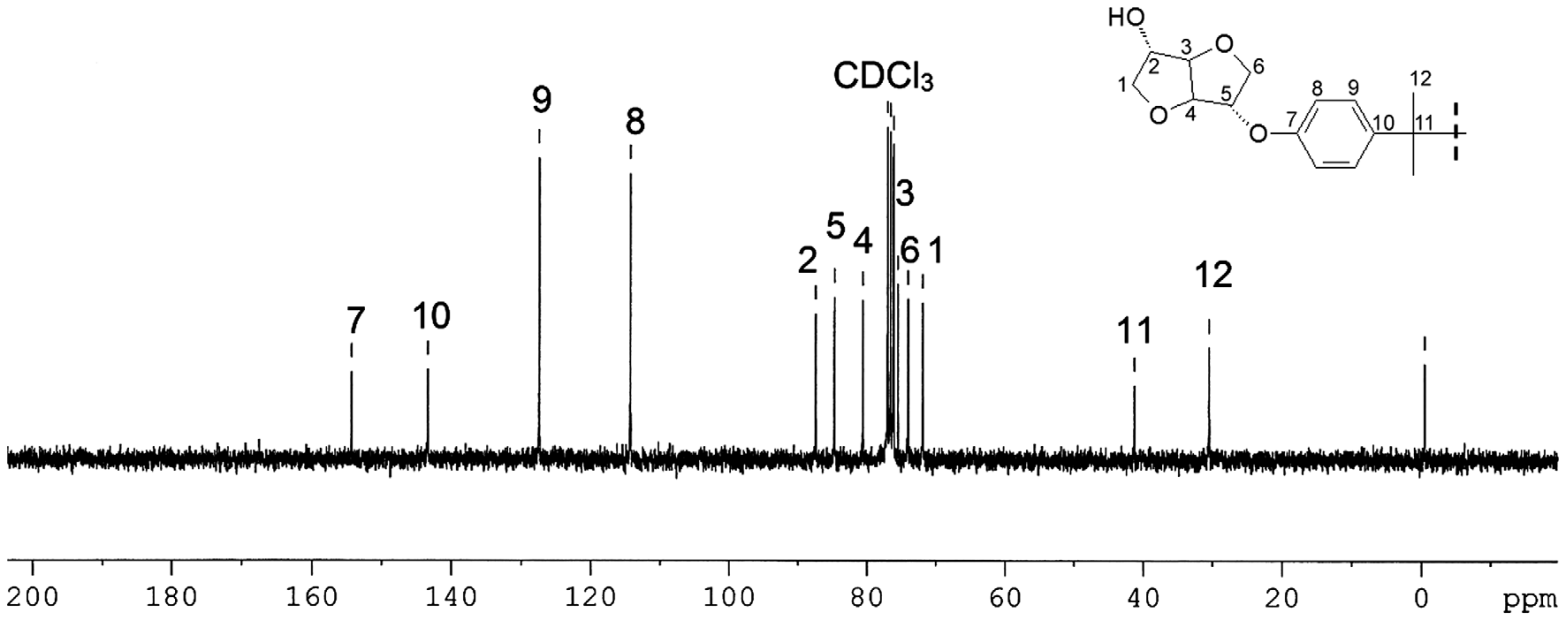
^13^C NMR spectrum of the monomer **4a** (75 MHz, 293 K, CDCl_3_).

**Figure 4.**
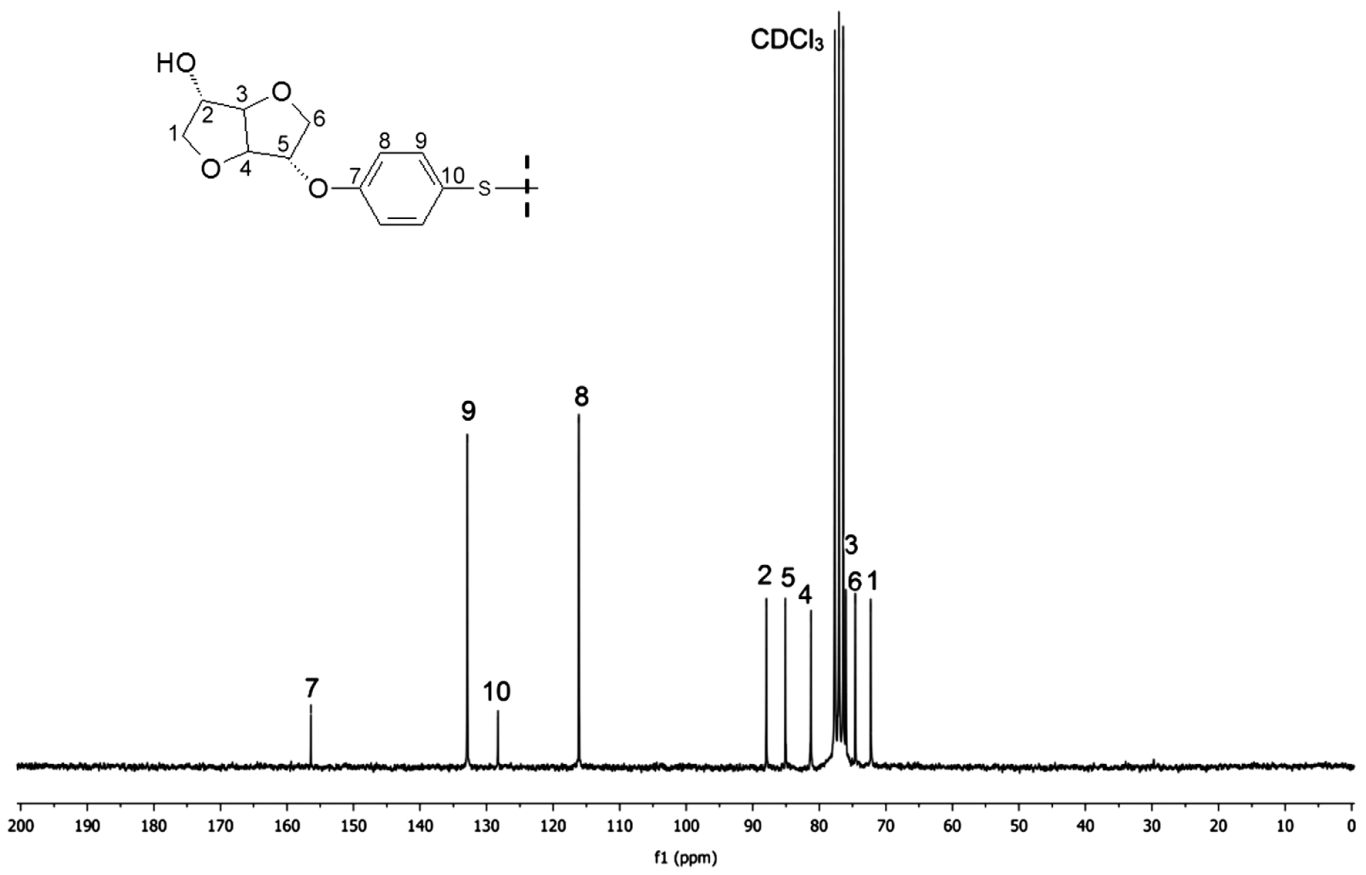
^13^C NMR spectra of the monomer **4b** (75 MHz, 293 K, CDCl_3_).

Figures 1 and 2 display the ^1^H NMR spectra of the two synthesized monomers **4a** and **4b**. The peaks between 6.81 and 7.12 ppm for **4a** and 6.88 and 7.28 for **4b** were consistent with the aromatic protons. The isosorbide protons appeared in the 3.87–4.74 ppm range for **4a** and in the 3.93–4.80 ppm range for the diol **4b**. The isosorbide *exo* hydroxyl groups showed a peak at 1.97 ppm for the monomer **4a**, and at 1.85 ppm for the diol **4b**. The BPA methyl groups engendered a singlet at 1.6 ppm. The Figures 3 and 4 report the ^13^C NMR spectra of the two diols. For the diol **4a**, we observed 4 carbon signals at 154.80, 143.58, 128.02 and 115.16 ppm which were attributed to aromatic carbons. Six peaks appeared at 88.21, 85.10, 81.08, 75.10, 74.69 and 71.83 ppm which were related to isosorbide carbons. The aliphatic carbons showed two peaks at 42.1 and 31 ppm. In the ^13^C NMR spectrum of the diol **4b**, 4 peaks were observed at 156.39, 132.89, 128.28 and 116.15 corresponding to the aromatic ring. 6 peaks located at 87.94, 85.02, 81.39, 76.00, 74.60 and 72.27 were characteristic of isosorbide carbons.

#### 3.2.2. FT-IR characterization

**Figure 5.**
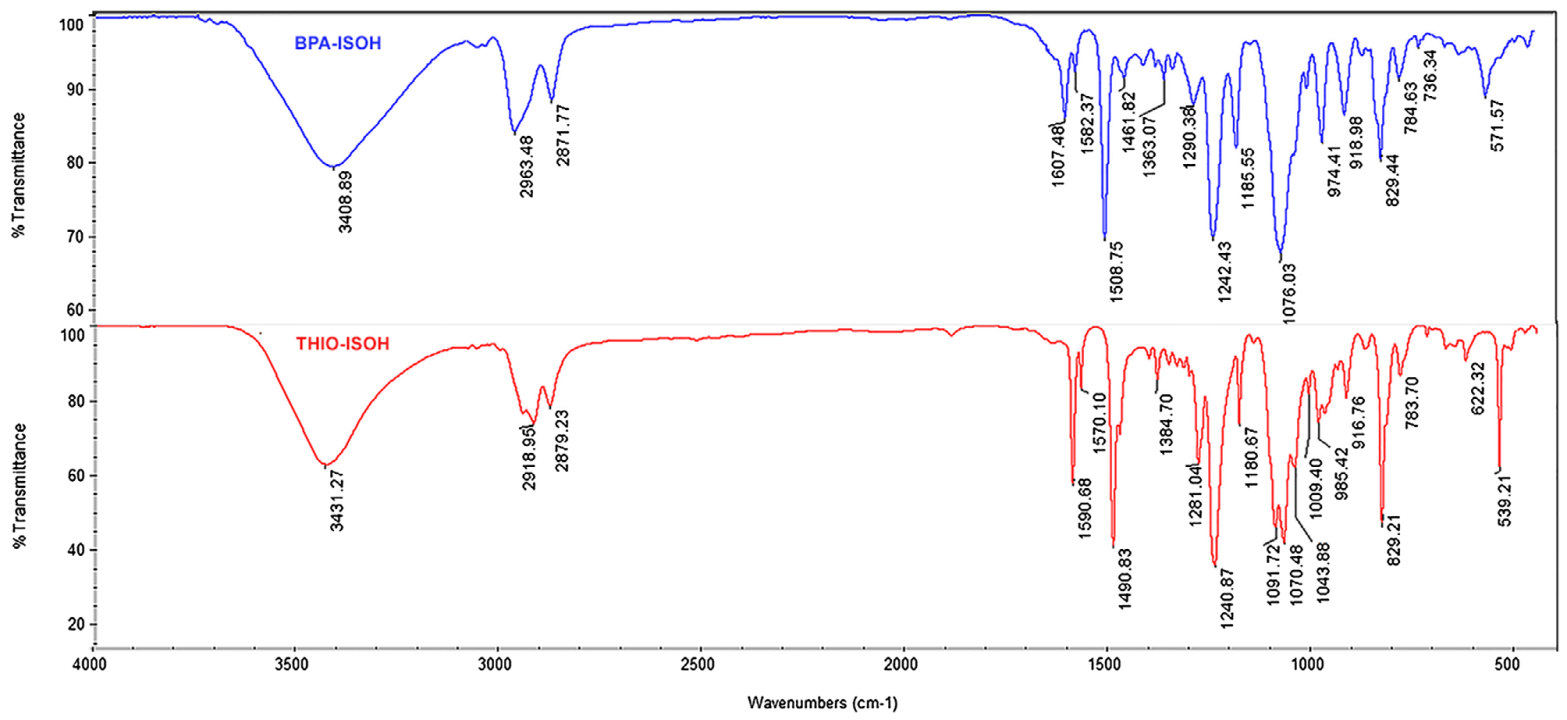
FT-IR spectra of the two diols **4a (BPA-ISOH)** and **4b (THIO-ISOH)**.

Figure 5 displays the FT-IR spectrum of the new diols **4a** and **4b** which exhibited the characteristic bands related to the two monomers. A broad band assigned to stretching vibration of the hydroxyl group was observed for all monomers between 3200 and 3600 cm^−1^, which confirmed the departure of the tosyl group and the accomplishment of the detosylation reaction. The stretching band of aromatic C–H was detected at 3050 cm^−1^ as well as the peak at 825 cm^−1^ was attributed to C–H of aromatic part; peak around 1490 cm^−1^ was assigned to the C = C stretching of aromatic part. Two bands at 2960 cm^−1^, 2865 cm^−1^ were attributed to aliphatic C–H stretching. Peak at 1075 cm^−1^ corresponded to stretching vibration of (C–O–C) associated to isosorbide and an absorption band observed at 1185 cm^−1^ was assigned to the stretching vibration of (C–O) adjacent to the alcohol function. All the peaks confirmed the new monomers structures.

#### 3.2.3. Thermal properties of the monomers

**Figure 6.**
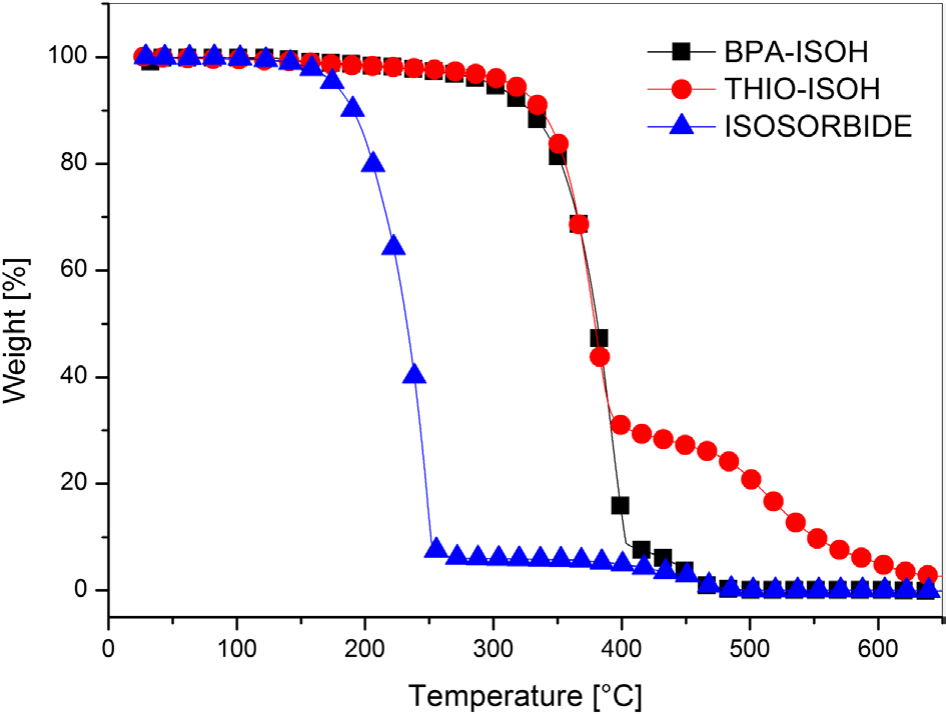
TGA thermograms of the monomers **4a** (BPA-ISOH), **4b** (THIO-ISOH) and isosorbide under nitrogen atmosphere.

**Figure 7.**
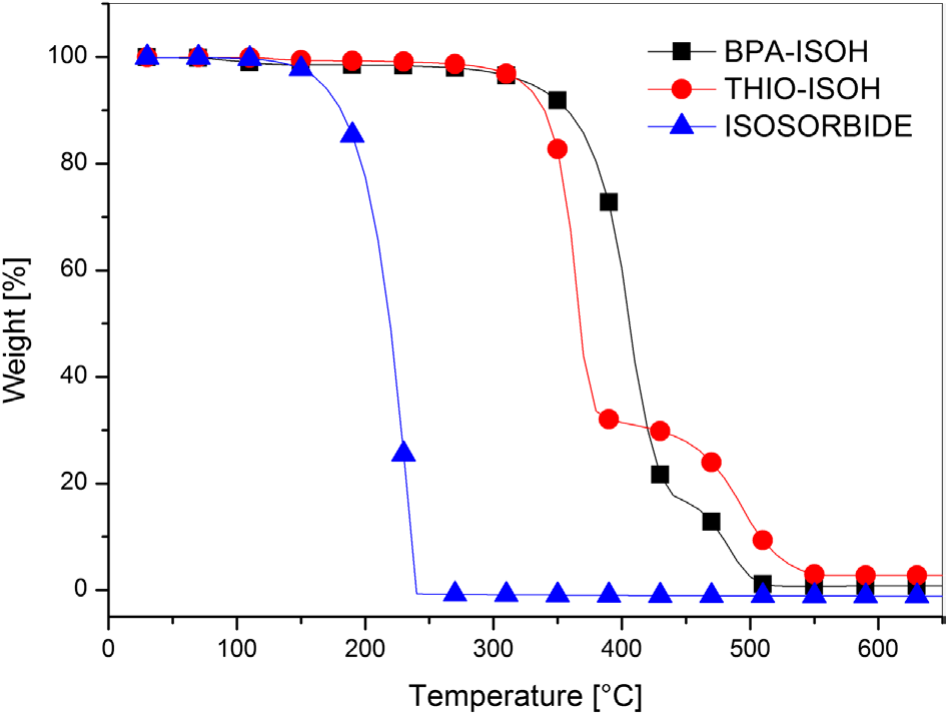
TGA thermograms of the monomers **4a** (BPA-ISOH), **4b** (THIO-ISOH) and isosorbide under air atmosphere.

Thermal properties of synthesized diols were studied by thermogravimetry analyses (TGA) under nitrogen and air atmosphere. The TGA curves in nitrogen (Figure 6) and air (Figure 7) of both monomers **4a** (BPA-ISOH) and **4b** (THIO-ISOH) showed no significant weight loss up to 400 °C in nitrogen and 350 °C in air; after that, they underwent a two-stage thermal degradation. The thermal stability of **4a** and **4b** was much higher than that of the parent isosorbide, both in air and nitrogen, and this could be mainly due to the presence of the aromatic unit (thiodiphenol and bisphenol A). Indeed, it is known that the thermal stability of polyols comprising aromatic structure is higher than that of aliphatic and cycloaliphatic polyols [40,41]; this is due to the formation of char, promoted by aromatics, which hinders the thermal degradation phenomena [40,41]. BPA-ISOH showed a slightly higher thermal stability in air than in nitrogen (Figure S7 in supplementary information), in agreement with Jang et al. [42]. Indeed, they reported that oxygen may facilitate branching of the phenyl radicals produced in the early stage of degradation leading to the formation of an intermediate char. This char interfered with the mass transfer through the surface of degrading material and thus, at the beginning of degradation, in air a slower mass loss rate was observed than in nitrogen. THIO-ISOH (Figure S8 in supplementary information) showed the same thermal stability both in air and nitrogen up to 500 °C while, at higher temperature, a lower weight was retained in air probably owing to the oxidation of C–S bonding with formation of SO_2_ and some sulfate derivatives [28,43]. When comparing the two diols **4a** and **4b**, it can be observed that the diol THIO-ISOH retained higher weight than BPA-ISOH at temperature higher than 400 °C in nitrogen and 450 °C in air; also the char residue at high temperature (600 °C) was higher for THIO-ISOH than BPA-ISOH. This could be ascribed to a greater promotion of char formation by the sulphur in THIO-ISOH, which hindered the formation of volatile degradation products [28]; in air sulphur could also provide an additional protection at high temperature in the form of sulfate [43].

**Figure 8.**
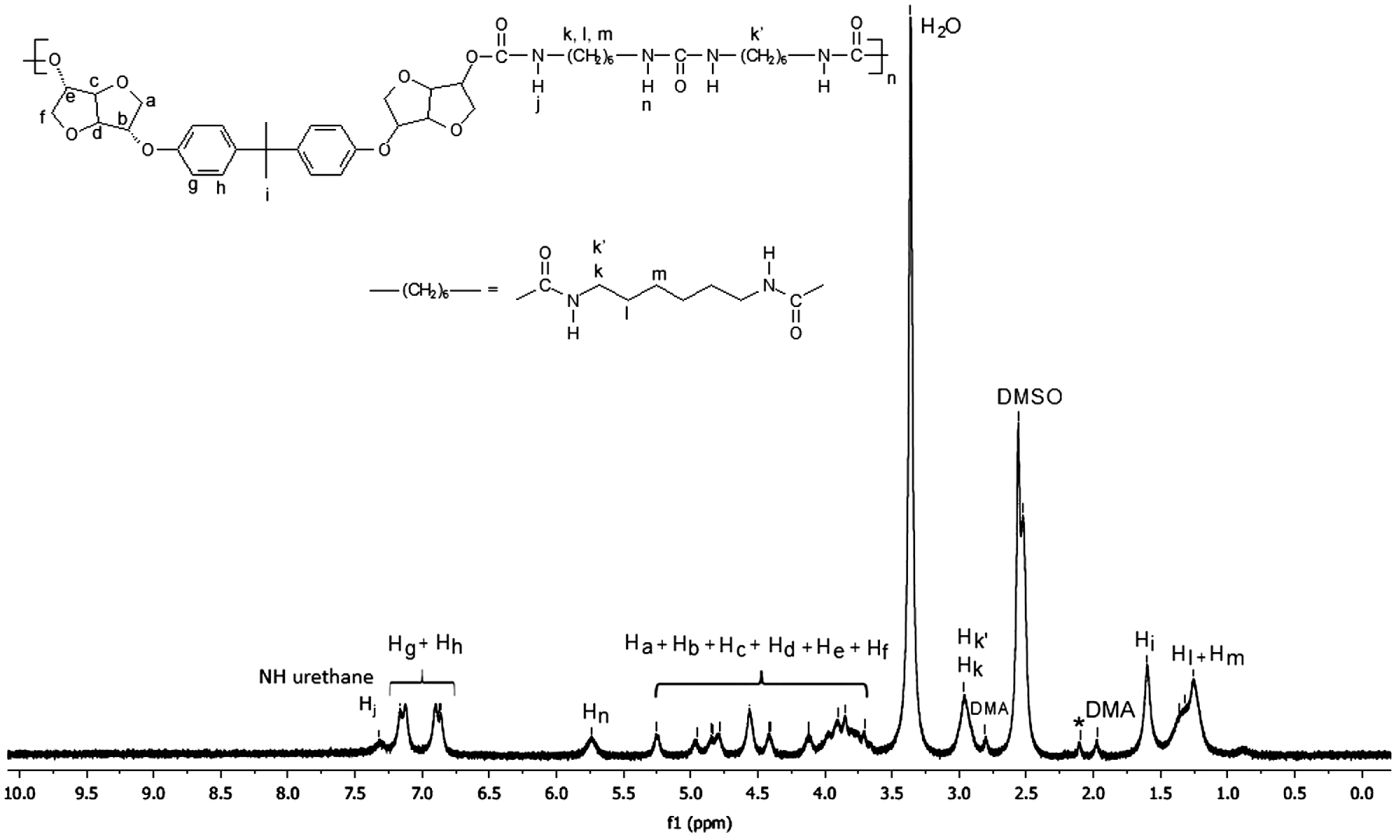
^1^H NMR spectrum (DMSO-d_6_) of the polyurethane **PU(HDI)BPA**.

**Figure 9.**
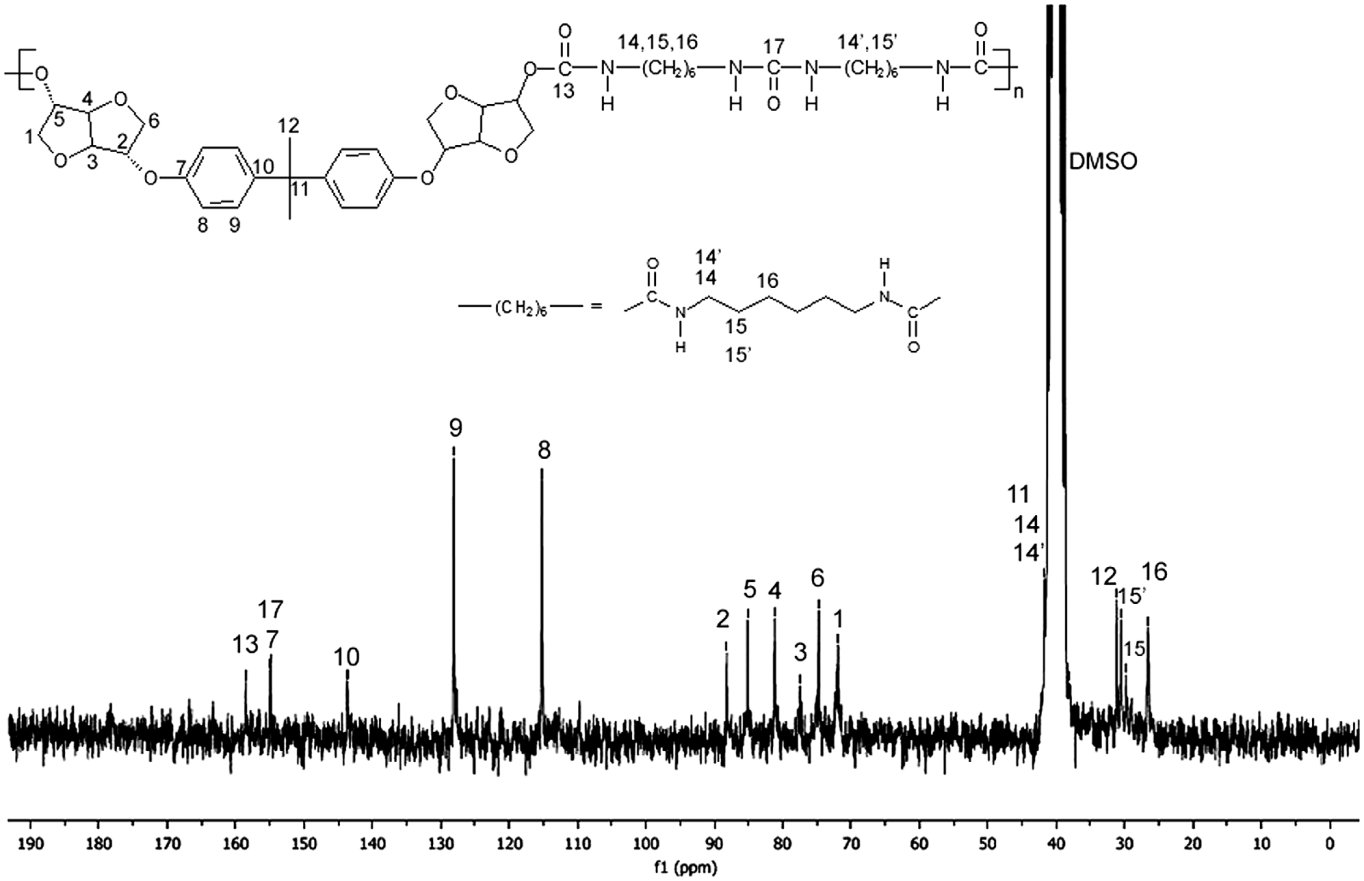
^13^C NMR spectrum (DMSO-d_6_) of the polyurethane **PU(HDI)BPA**.

**Figure 10.**
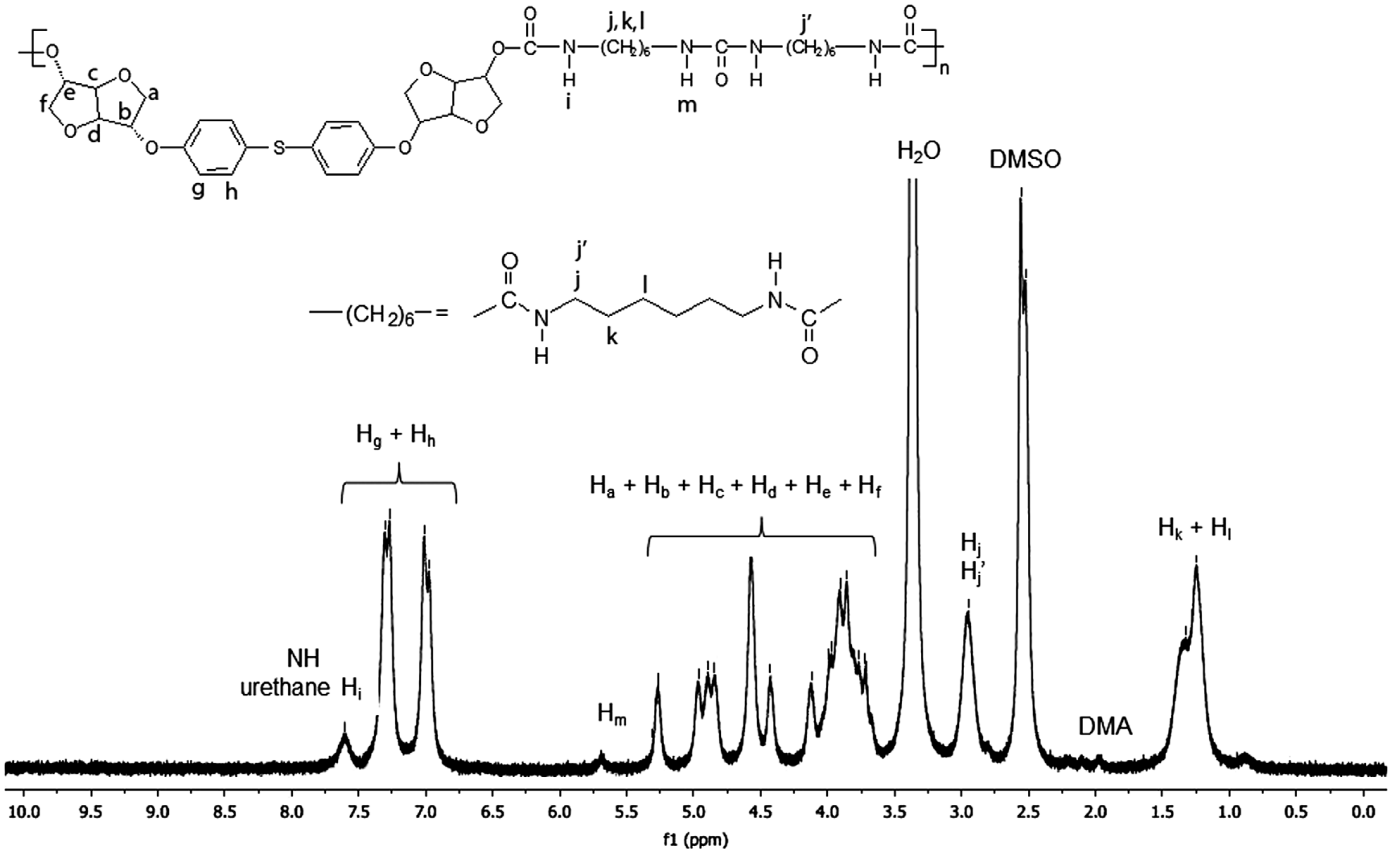
^1^H NMR spectrum (DMSO-d_6_) of the polyurethane **PU(HDI)THIO**.

**Figure 11.**
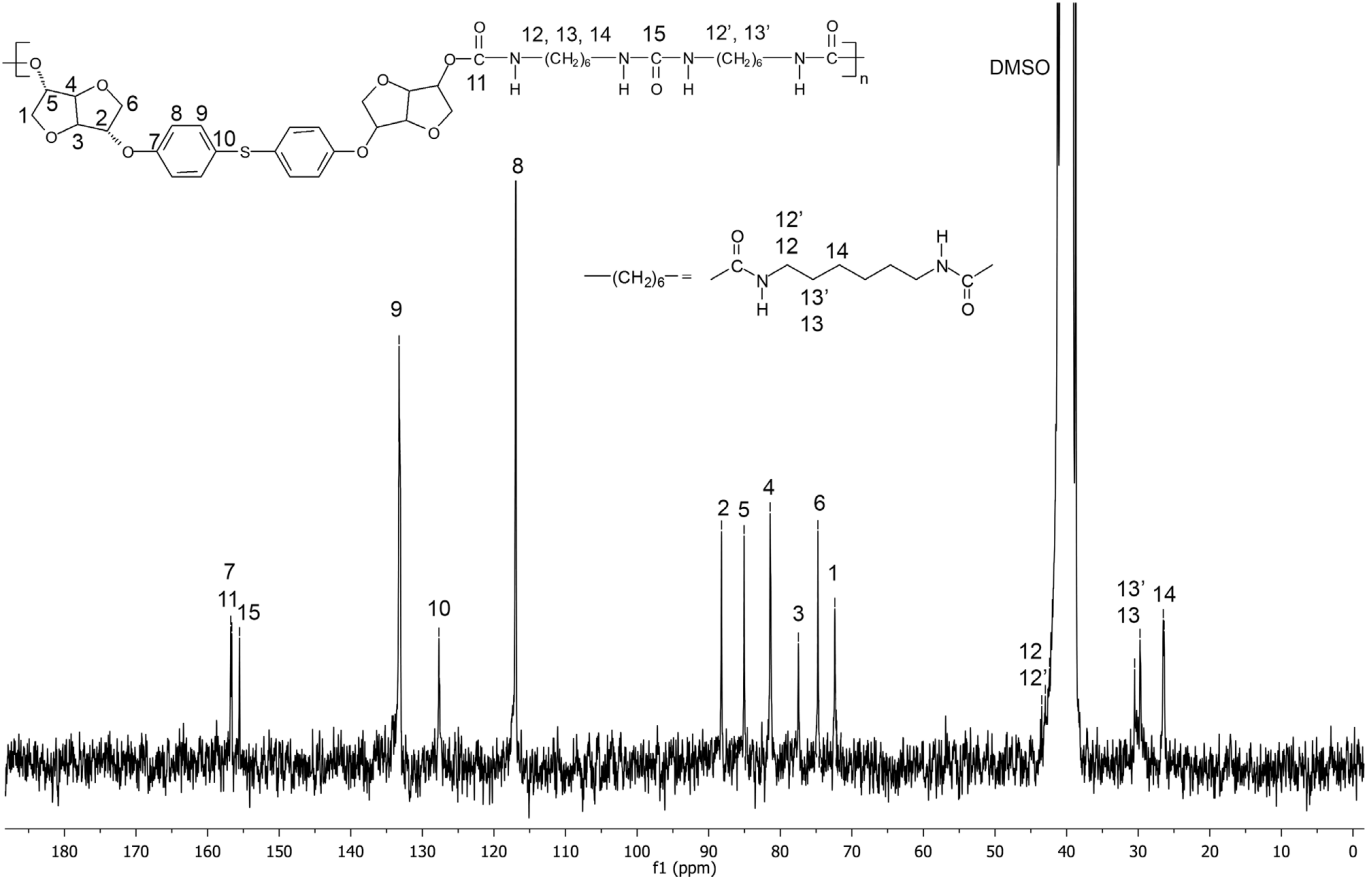
^13^C NMR spectrum (DMSO-d_6_) of the polyurethane **PU(HDI)THIO**.

**Figure 12.**
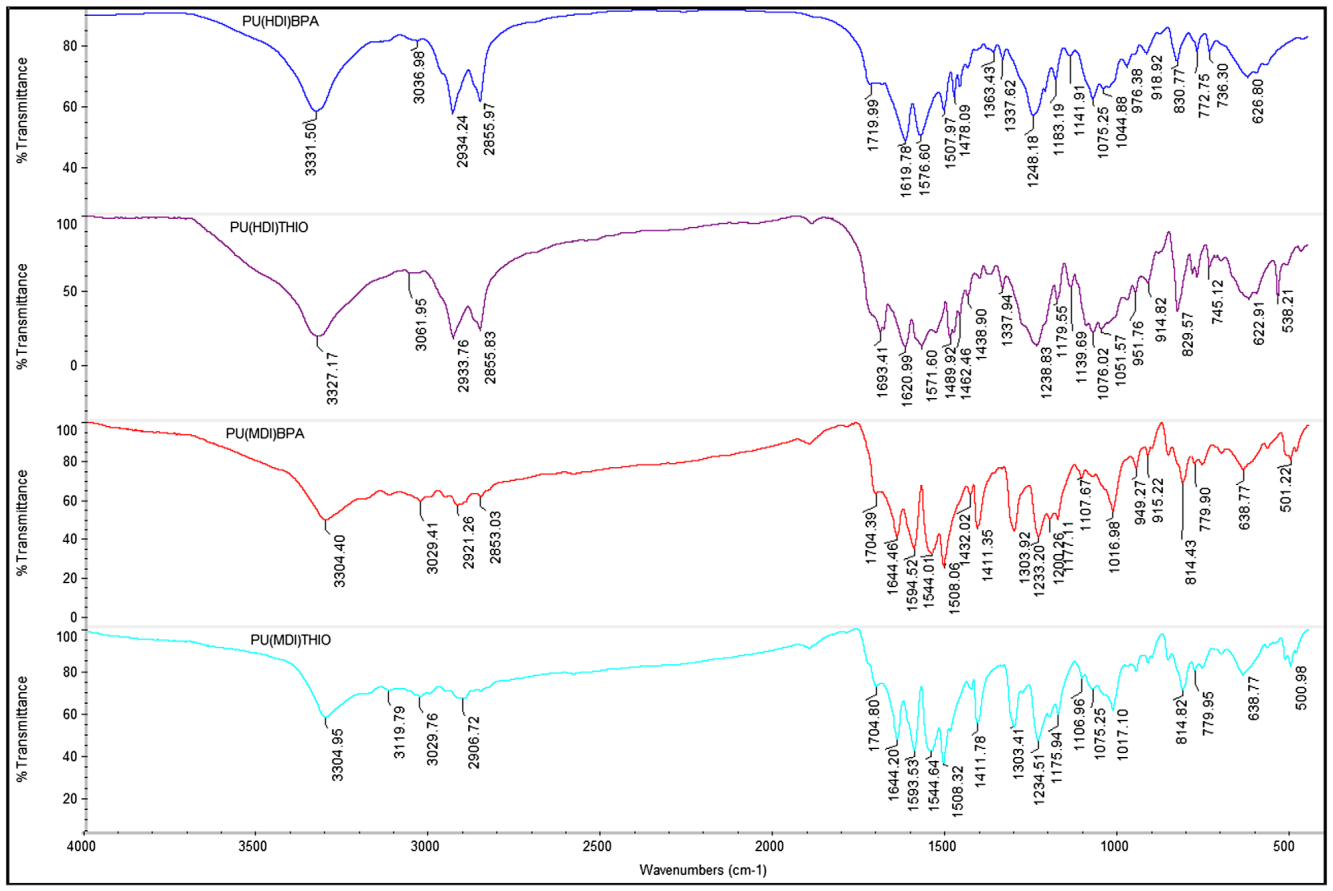
FTIR spectra of the polyurethanes **PU(MDI)BPA**, **PU(HDI)BPA**, **PU(MDI)THIO** and **PU(HDI)THIO**.

**Figure 13.**
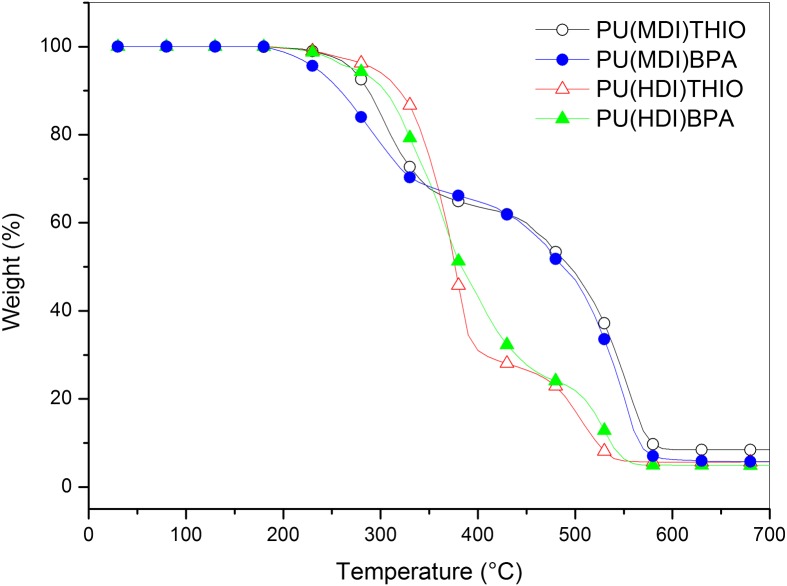
TGA traces of polyurethanes **PU(MDI)BPA**, **PU(HDI)BPA**, **PU(MDI)THIO** and **PU(HDI)THIO** under air atmosphere.

**Figure 14.**
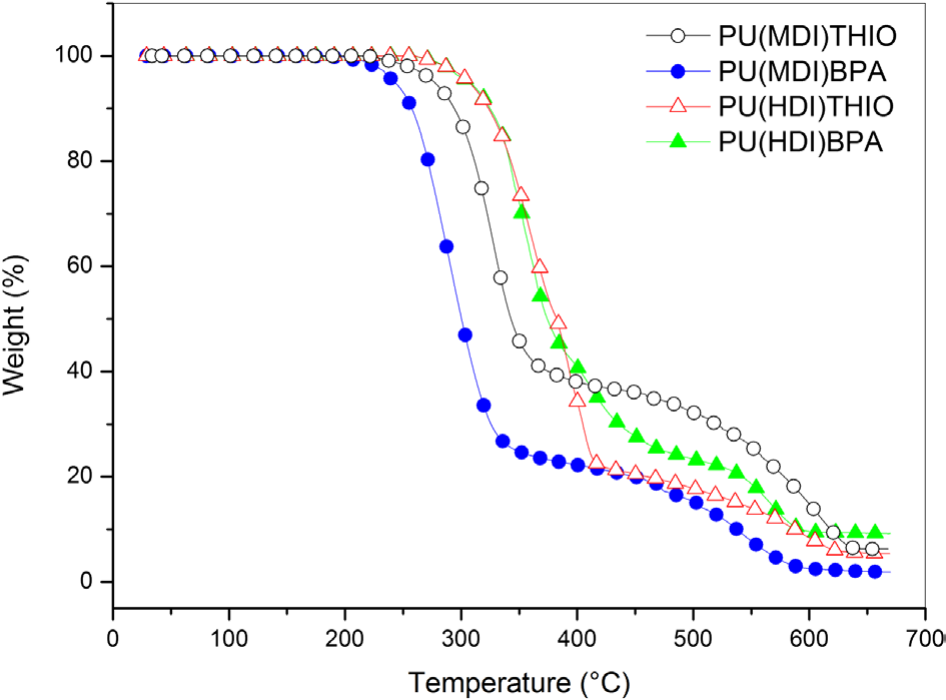
TGA traces of polyurethanes **PU(MDI)BPA**, **PU(HDI)BPA**, **PU(MDI)THIO** and **PU(HDI)THIO** under nitrogen atmosphere.

### 3.3. Polymerization of the diol (4a) with MDI and HDI

**Scheme 2.**
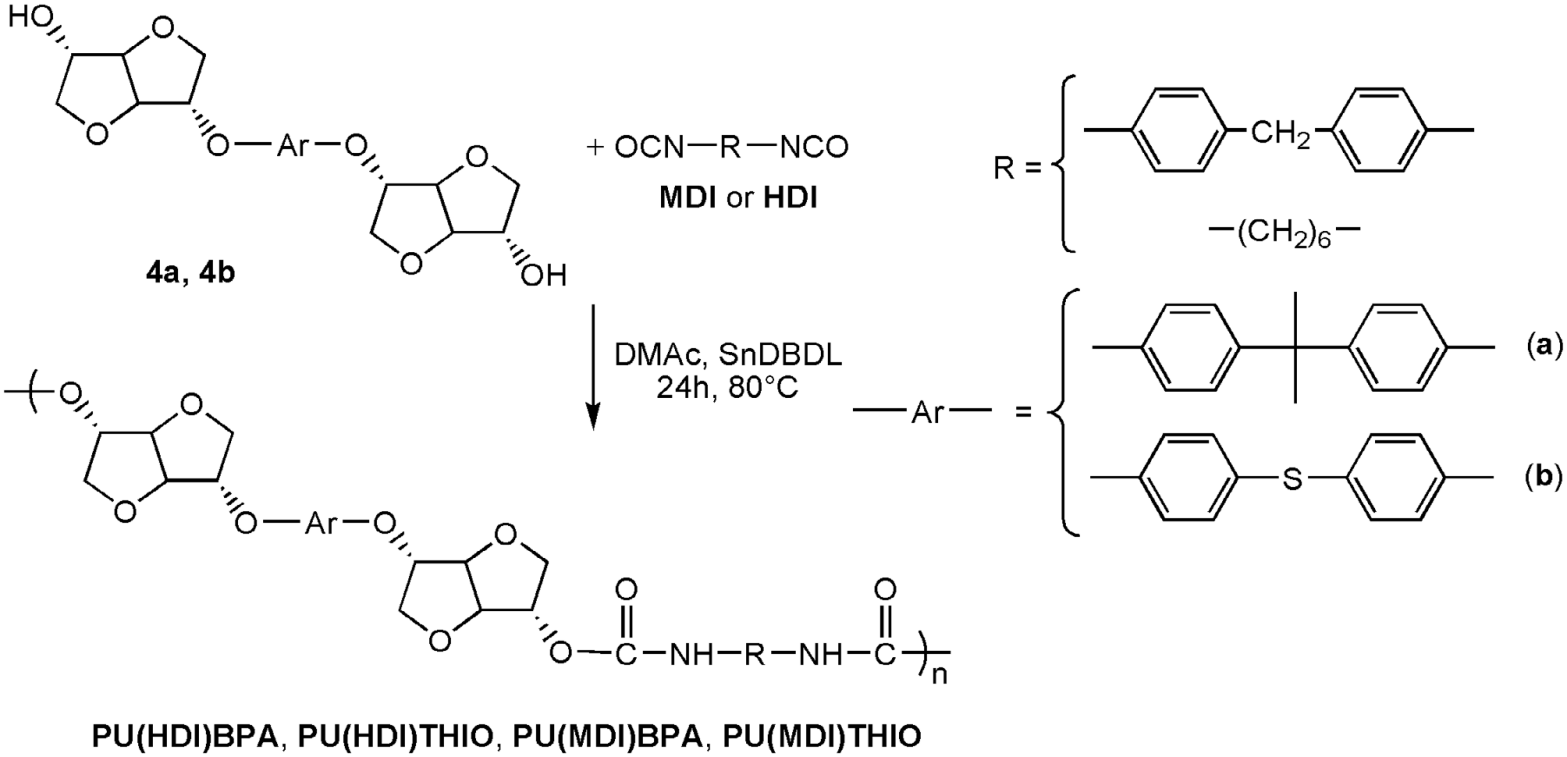
Polymerization of the diols **(4a)** and (**4b**) with the diisocyanate MDI and HDI.

In order to prove the utility of diols **4a** and **4b** as building block in polymer materials, we carried out the polymerization of the monomer **4a** and **4b** to synthesize four polyurethanes. These were synthesized by polymerisation reaction in solution of the diols **4a** and **4b** and two kinds of difunctional isocyanates, HDI and 4,4′–diphenylmethane diisocyanate (MDI), using dibutyl dilaurate (SnDBDL) as catalyst (Scheme 2). The reaction was carried out in DMAc at 80 °C for 24 h [44]. The polyurethanes were obtained by precipitation in cold methanol and thereafter drying under vacuum at room temperature. ^1^H and 13C NMR spectra (Figures 8–11 and Figures S9-S12 found in the supplementary information) recorded in DMSO-d6 of the synthesized polyurethanes PU(HDI)THIO, PU(HDI)BPA, PU(MDI)BPA and PU(MDI)THIO allowed us to determine and attribute all the signals. The 1H NMR spectra of 4a and 4b-based TPUs using MDI as isocyanate displayed a characteristic signal of urethane moiety (–NHCO–) at 9.76 and 9.74 ppm, respectively, confirming the formation of the new polyurethane. This signal appeared as a singlet (and not as doublet) which confirmed that the diols BPA-ISOH and THIO-ISOH, prepared from isosorbide, have two equivalent hydroxyl functions in exo–exo position [44]. This result was confirmed and correlated with the observation of 13C NMR spectrum as the carbonyl function of urethane group (-CO-NH-) appeared as one signal only, for both TPUs, at 153.03 ppm (it did not appear as two signals as in the case of isosorbide which has two non-equivalent hydroxyl groups).

**Scheme 3.**
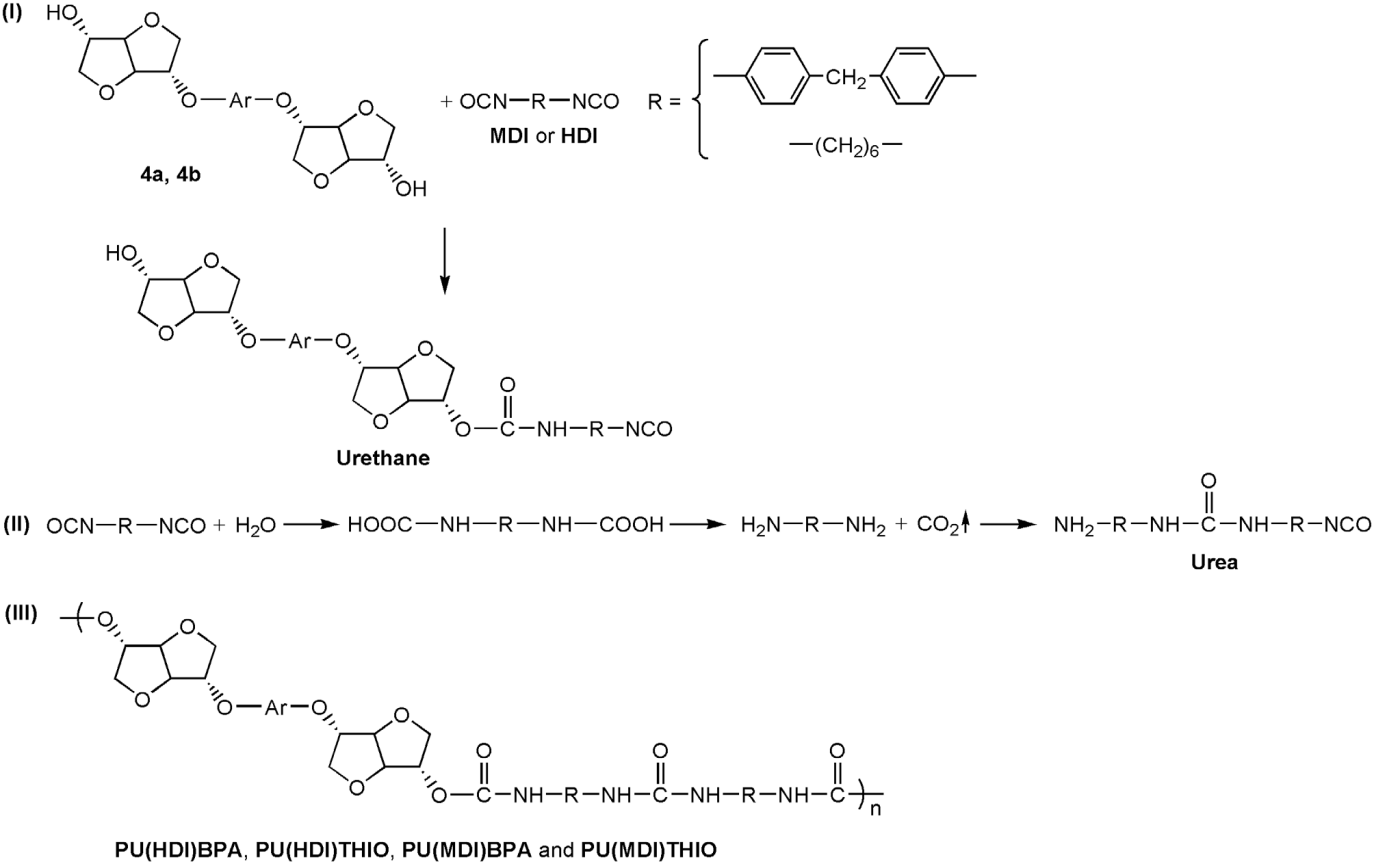
Mechanism of the reaction between the monomers **4a** (BPA-ISOH) or **4b** (THIO-ISOH) and MDI or HDI.

Beside the characteristic protons of polyurethanes, monomers BPA-ISOH and THIO-ISOH, 4,4′–diphenylmethane diisocyanate (MDI) and urethane group, an additional signal at 8.55 ppm was observed in the spectra, which confirmed the existence of urea group in the polymer structure. The analysis of 13C NMR spectrum of the polyurethane PU(MDI)BPA and PU(MDI)THIO, revealed in the region (114–160 ppm) the existence of a signal at 147.07 ppm corresponding to carbonyl of urea group. In this regard, and in connection with our work, the study by Beldi et al. [44] described the synthesis of new cyclic and non-cyclic poly-(ether-urethane)s based on isosorbide. They reported that the peak observed at 8.52 ppm was related to aromatic urea function. This result was consistent with our work and allowed us to correctly identify all peaks and signals founds in its ^1^H and ^13^C NMR spectra (Figures S5, S6, S7 and S8). The polyurethanes structure, which was in agreement with the result of NMR spectra, can be represented as shown below (Scheme 3). The reaction mechanism to obtain the structure of **PU(MDI)BPA** and **PU(MDI)THIO** containing urea aromatic group, which was in good correlation with the result obtained from ^1^H and ^13^C NMR spectra, is depicted in Scheme 3. The water involved in the reaction was due to humidity uptake by the diol before the reaction because of its high hygroscopicity. ^1^H NMR spectra of polyurethanes **PU(HDI)BPA** and **PU(HDI)THIO** (Figures 8 and 10) exhibited all the protons characteristic of HDI (H_k_, H_l_ and H_m_), of isosorbide (H_a_-H_f_) and of the urethane group (H_i_ and H_j_ at 7.32 ppm and at 7.51 ppm). Since the starting monomers **BPA-ISOH** and **THIO-ISOH** of the polyurethanes **PU(HDI)BPA** and **PU(HDI)THIO** had two equivalent hydroxyl groups in *exo* position, the signals of urethane moiety appeared as singlet at 7.32 ppm and at 7.51 ppm (and not a doublet as in the case of isosorbide due to its two non-equivalent hydroxyl groups). For the polyurethanes **PU(HDI)BPA** and **PU(HDI)THIO** obtained from HDI, aside from the characteristic peaks of urethane group, at 7.32 and 7.51 ppm in ^1^H NMR and, at 158.05 and 156.76 ppm in ^13^C NMR (Figures 9 and 11), respectively, two additional signals were detected corresponding to the urea group: one at 5.73 ppm (for both polyurethanes) in ^1^H NMR spectrum (related to the proton of urea group) whereas the second signal was observed at 154.97 ppm (derived from **4a**) and 155.53 ppm (derived from **4b**) in ^13^C NMR spectrum which is related to the carbon of the urea carbonyl. Therefore, the structure of the polyurethanes **PU(HDI)BPA** and **PU(HDI)THIO** should contain urea group. Based on this, we can assess that the polyurethanes derived from HDI and diols **4a** and **4b** were obtained through the same reaction mechanism as shown in the Scheme 3 (considering HDI instead of MDI). The FT-IR spectra of the four polymers (Figure 12) confirmed the presence of characteristic bands due to the formation of urethane linkages in the synthesized polyurethanes. These signals appeared for **PU(MDI)BPA** at 3304, 1705, 1644 and 1544 cm^−1^ which were assigned to NH, C = O of urethane and urea, and N–H of urethane, respectively. The same characteristic peaks were present for the others three polyurethanes **PU(HDI)BPA**, **PU(HDI)THIO** and **PU(HDI)THIO**. Due to their insolubility in THF, the average molecular weights of the polyurethanes prepared cannot be determined by SEC. However, intrinsic viscosity [*η*] of **PU(HDI)BPA**, **PU(HDI)THIO, PU(MDI)BPA** and **PU(MDI)THIO** has been measured at 25 °C using a mixture of phenol and tetrachloroethane and the results are reported in Table 6. The results obtained were in good agreement with those reported by Kayalvizhi et al. [45] and Lee et al. [46] for polyurethane polymers using the same solvent, therefore indicating the formation of polyurethane with medium-to-high molecular weights. Thermal properties of the synthesized polymers were investigated by differential scanning calorimetry (DSC) analysis with a heating rate of 10 °C/min. Glass transition temperature (*T*_*g*_) and melting temperature (*T*_*m*_) during the first and second heating are reported in Table 7. An example of DSC thermogram is reported in the supplementary information (Figure S13). *T*_*g*_ of HDI based polyurethane were similar to those reported for similar polymer structures [16,18,19,44]; similar results have been obtained also by Javni et al. [17] although they used, beside isosorbide, MDI and PTMG as soft segment. This is not surprising since in our HDI polyurethane, HDI played a role in enhancing chain flexibility probably in the same way that PTMG did in Javni’s polyurethanes. Indeed, it can be observed in Table 7 that *T*_*g*_ was significantly higher in MDI-polyurethanes than in their corresponding HDI-counterparts. These results were in agreement with those reported by other authors [16,18,19,44] and were highly expected owing to the increase in chain stiffness caused by the presence of the biphenyl unit in MDI. Obviuosly, for both HDI- and MDI-polyurethanes, also the rigidity of the diols used played a role in the *T*_*g*_ value. All the polyurethanes obtained were crystalline materials. During second heating, HDI-based polyurethane showed broad multiple melting peaks, presumably indicating the occurrence of crystallite size heterogeneities, as already reported by Marin et al. [16]. MDI-based polymers looked amorphous in the second scan. This may result from the proximity of the glass transition and melting temperatures as well as to the low chain flexibility. Similar behavior was observed by Cognet-Georjon et al. [47], who compared also the *T*_*g*_ between rigid polyurethanes based on isosorbide with various common diols and found that the glass transition temperature of the polymer obtained with isosorbide was strikingly above the values associated with all the others. This makes isosorbide and derived diols particularly interesting candidates for the chain extension of segmented polyurethanes, since the thermal stability of their hard segments would be greatly improved compared to classical systems. Moreover, the melting temperature obtained were not too high and these polymers should thus remain processable at reasonable temperatures. The thermal stability of the four synthesized polyurethanes **PU(MDI)BPA**, **PU(HDI)BPA**, **PU(MDI)THIO** and **PU(HDI)THIO** has been studied by thermogravimetric analysis (TGA) under air and nitrogen atmosphere. The TGA thermograms are reported Figures 13 and 14. It can be observed that the thermal stability was higher for HDI-TPUs up to 400 °C (i.e., during the first step of degradation) probably because of the slightly higher molecular weight of these polymers with respect their MDI-counterparts, as it can be inferred by intrinsic viscosity measurements. In this step, indeed, depolymerisation, through urethane bond scission, took place [48]. In the second step, where decomposition of isocyanate and aromatic compounds took place [48], the major role in determing thermal stability was played by the aromaticity of the polymers, so MDI-polyurethanes showed higher thermal stability. This effect was more pronounced in air than in nitrogen. In order to compare the thermal stability of the polyurethanes developed in this work with other similar structures already reported in the literature, some decomposition parameters are reported in Table 8 where *T*_5%_ and *T*_10%_ are the temperatures at which 5 or 10% of weight has been lost and ^max^ *T*_*d*_ states for the temperatures at which maximum degradation rate takes place for every step of degradation. Considering our samples, it seemed that the presence of sulphur in THIO samples was effective in delaying somewhat the onset of degradation (*T*_5%_) as well as slowing the degradation process since the maximum degradation rate took place at higher temperatures. With respect the data we found in literature, the HDI-based polyurethanes synthesized according to the procedure proposed here showed an onset temperature (*T*_5%_), both in nitrogen or air, up to 40 °C higher (**PU(HDI)BPA** vs **PU(HDI)IS** [18] or **PU(HDI)IS** [19]) while no significant difference has been revealed for MDI-based polymers. Also the temperatures at which maximum degradation rate took place (^max^ *T*_*d*_) were higher, mainly for the second degradation step, and this was probably related to the aromatic nature of the comonomers (BPA and THIO) used in diols synthesis; some contributions of sulphur could also be important.

**Table 6.** Intrinsic viscosity of the polyurethanes.

Sample	**PU(HDI)BPA**	**PU(HDI)THIO**	**PU(MDI)BPA**	**PU(MDI)THIO**
*η* [dl/g]	0.43	0.46	0.41	0.42

**Table 7.** Glass transition and melting temperatures of the polyurethanes.

Sample	*T*_*g*_ [°C]	*T*_*m*_ [°C]	
		1st heating	2nd heating
**PU(MDI)THIO**	175	228	–
**PU(MDI)BPA**	183	200	–
**PU(HDI)THIO**	52	267	260/267
**PU(HDI)BPA**	61	283	270/279

**Table 8.** Decomposition parameters of the polyurethanes compared with data taken from Refs. [16,18,19].

	Nitrogen			Air		
	*T*_5%_[°C]	*T*_10%_[°C]	^max^*T*_*d*_[°C]	*T*_5%_[°C]	*T*_10%_[°C]	^max^*T*_*d*_[°C]
Sample						
**PU(MDI)THIO**	281	297	331/609	270	288	305/555
**PU(MDI)BPA**	247	260	290/544	235	258	341/596
**PU(HDI)THIO**	307	326	380/590	296	321	378/502
**PU(HDI)BPA**	313	325	359/549	276	308	362/527
**PU(HDI)IS** [19]	–	–	–	260	298	335/396
**PU(HDI)IS** [16]	296	–	342/471	–	–	–
**PU(HDI)IS** [18]	277	–		–	–	–
**PU(MDI)IS** [18]	284	–	–	–	–	–
**PU(MDI)IS** [16]	297	–	346/375	–	–	–

## 4. Conclusions

We successfully synthesized for the first time two new chiral *exo*–*exo* configured isoidide-derived diols from isosorbide. The molecular structures were confirmed by ^1^H NMR, ^13^C NMR and FTIR. We optimized the O-alkylation reaction, which was the second step in the synthesis, under conventional heating and using microwave irradiations. We proved the efficiency of microwave activation as an efficient, fast and simple route to O-alkylation reaction: it dramatically shortened the reaction time as well as improves yield. The diols synthesized showed significantly improved thermal stability with respect to the parent isosorbide and can be used in polyurethane synthesis. The thermoplastic polyurethanes thus obtained showed semi-crystalline behaviour and very good thermal stability. It was also shown that the introduction of sulphur in the polyurethane backbone was effective in delaying somewhat the onset of degradation as well as the degradation process. It seemed also that the derived diols can be interesting candidates for the chain extension of segmented polyurethanes, since the thermal stability of their hard segments would be greatly improved compared to classical systems.

## Supplemental data

Supplemental data for this article can be accessed at https://doi.org/10.1080/15685551.2017.1395502

## Funding

This work was financially supported by the Ministry of Higher Education and Scientific Research of Tunisia.

## Disclosure statement

No potential conflict of interest was reported by the authors.

## Supplementary Material

TDMP_1395502_Supplementary_Material.docx
