# Supplementary material for: Microwave-assisted synthesis of isosorbide-derived diols for the preparation of thermally stable thermoplastic polyurethane
Source: Des Monomers Polym. 2017 Nov 13;20(1):547–63. doi: 10.1080/15685551.2017.1395502 (PMC5812175; doi:10.1080/15685551.2017.1395502)
Supplement: TDMP_1395502_Supplementary_Material.docx [file TDMP_A_1395502_SM7123.docx]

**Microwave-assisted synthesis of isosorbide-derived diols for the preparation of thermally stable thermoplastic polyurethane**

Nejib Kasmi^a^, Martina Roso^b^, Nadia Hammami^a^, Mustapha Majdoub^a^, Carlo Boaretti^b^, Paolo Sgarbossa^b^, Chiara Vianello^b^, Giuseppe Maschio^b^, Michele Modesti^b^, Alessandra Lorenzetti^b,^*

^a^ Laboratory of Interfaces and Advanced Materials (LIMA), Faculty of Sciences of Monastir – Boulevard of the Environment, University of Monastir, Monastir, Tunisia

^b^ Department of Industrial Engineering & INSTM UdR Padova, University of Padova, Padova, Italy

**Correspondence to* Alessandra Lorenzetti. Tel.: +39 049 8275556; Fax: +39 049 8275555.

E-mail address: [alessandra.lorenzetti@unipd.it](mailto:alessandra.lorenzetti@unipd.it)

**Index for Supporting Information**

**^1^H and ^13^C NMR spectra of compound (2) Figures S1-S2**

**^1^H and ^13^C NMR spectra of compound (3a) Figures S3-S4**

**^1^H and ^13^C NMR spectra of compound (3b) Figures S5-S6**

**TGA in air and nitrogen of compound (4a) and (4b) Figures S7-S8**

**^1^H and ^13^C NMR spectra of polyurethane PU(MDI)BPA Figures S9-S10**

**^1^H and ^13^C NMR spectra of polyurethane PU(MDI)THIO Figures S11-S12**

**DSC thermogram of** **PU(HDI)BPA Figure S13**

Compound **2**


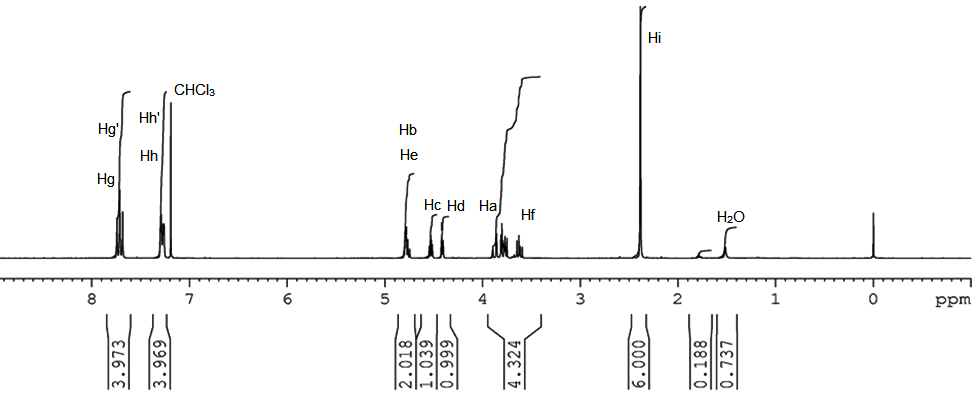


**Figure S1.** ^1^H NMR spectrum of ditosylated isosorbide **2** (300 MHz, 293 K, CDCl_3_)

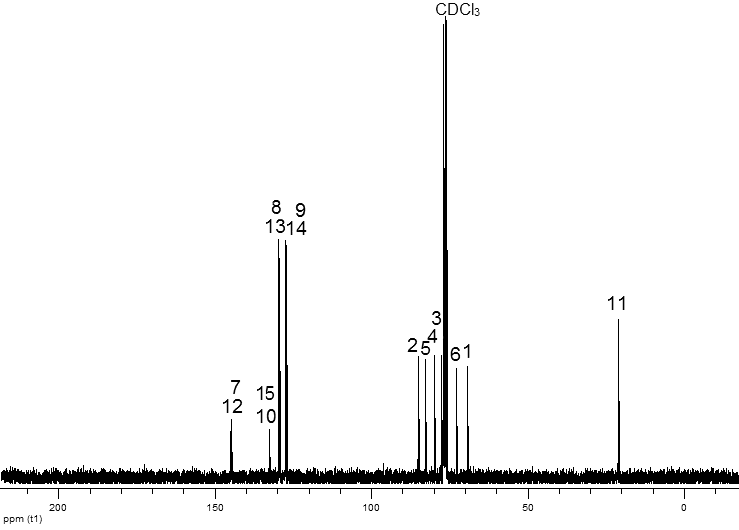


**Figure S2.** ^13^C NMR spectrum of ditosylated isosorbide **2** (in CDCl_3_)

Compound **BPA-ISDT** (**3a**)

#
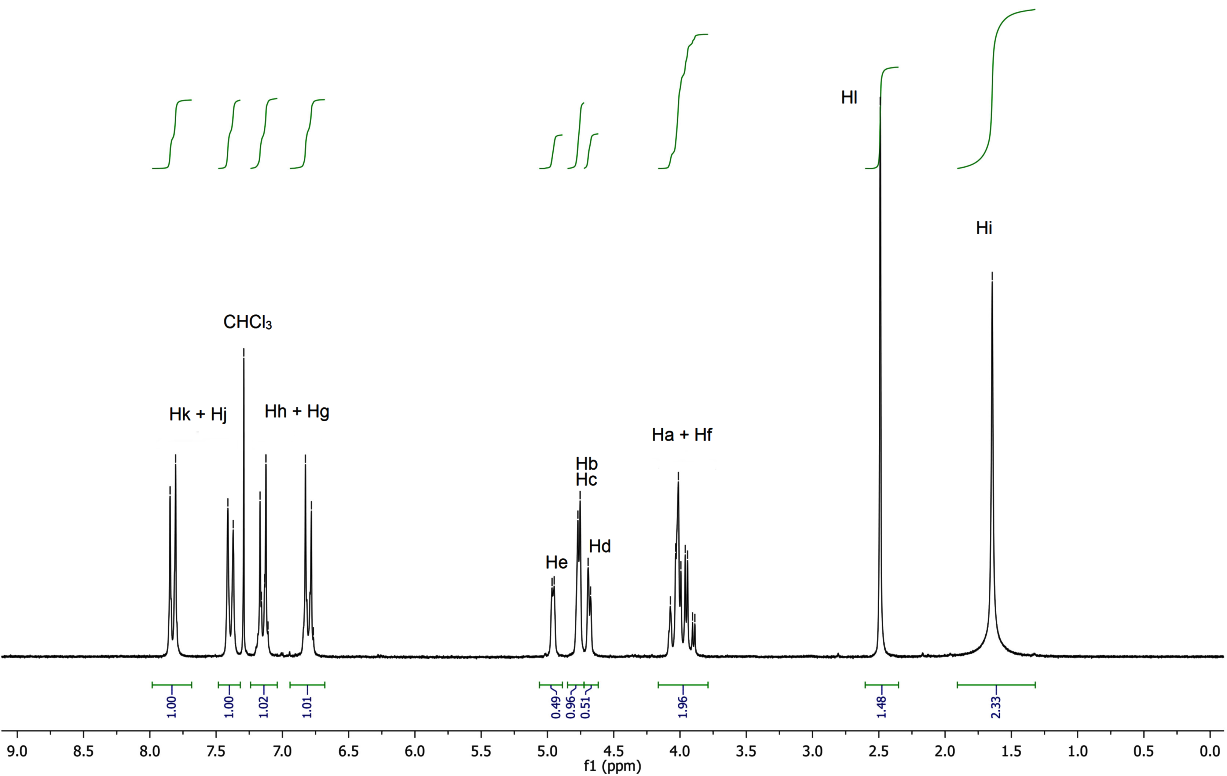


# Figure S3. ^1^H NMR spectrum of the intermediate BPA-ISDT (3a) (in CDCl_3_)

#

#
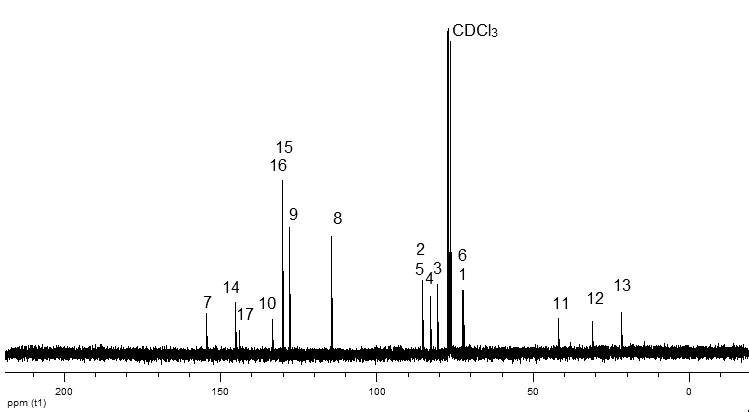


**Figure S4.** ^13^C NMR spectrum of the intermediate **BPA-ISDT** (**3a**) (in CDCl_3_)

Compound **THIO-ISDT** (**3b**)

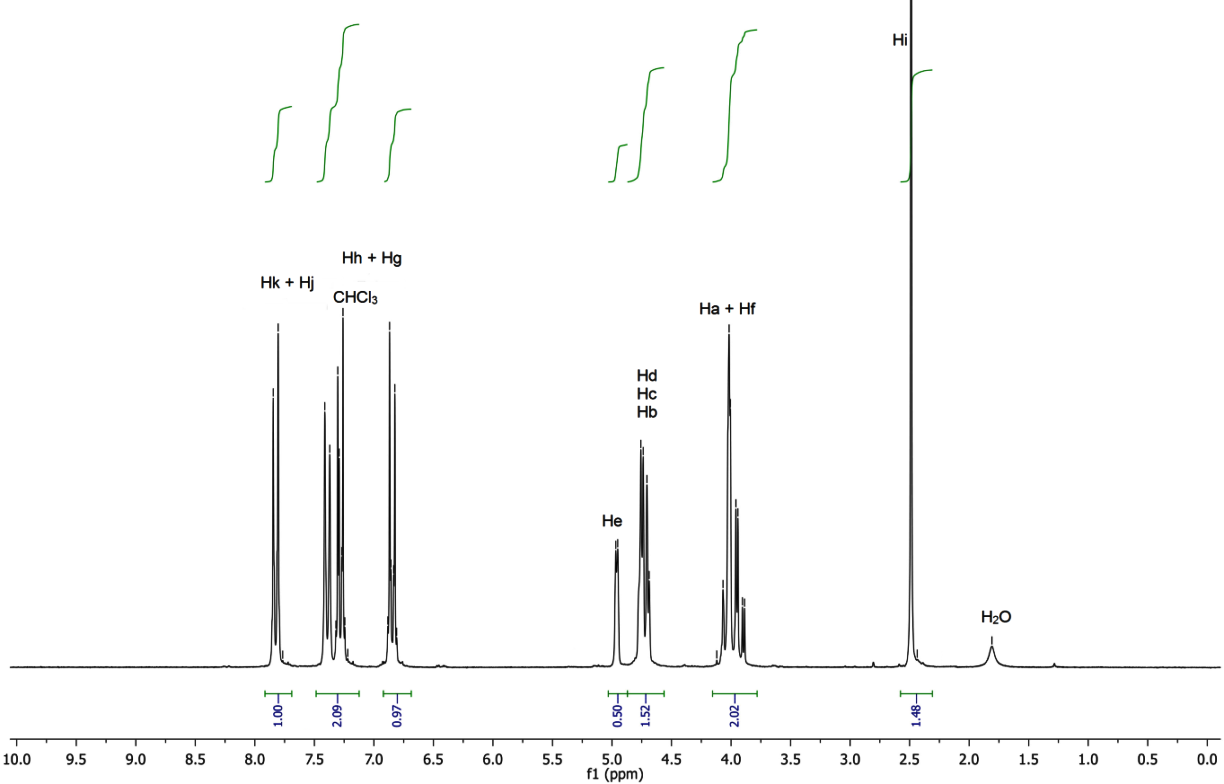


# Figure S5. ^1^H NMR spectrum of the intermediate THIO-ISDT (3b) (in CDCl_3_)

#

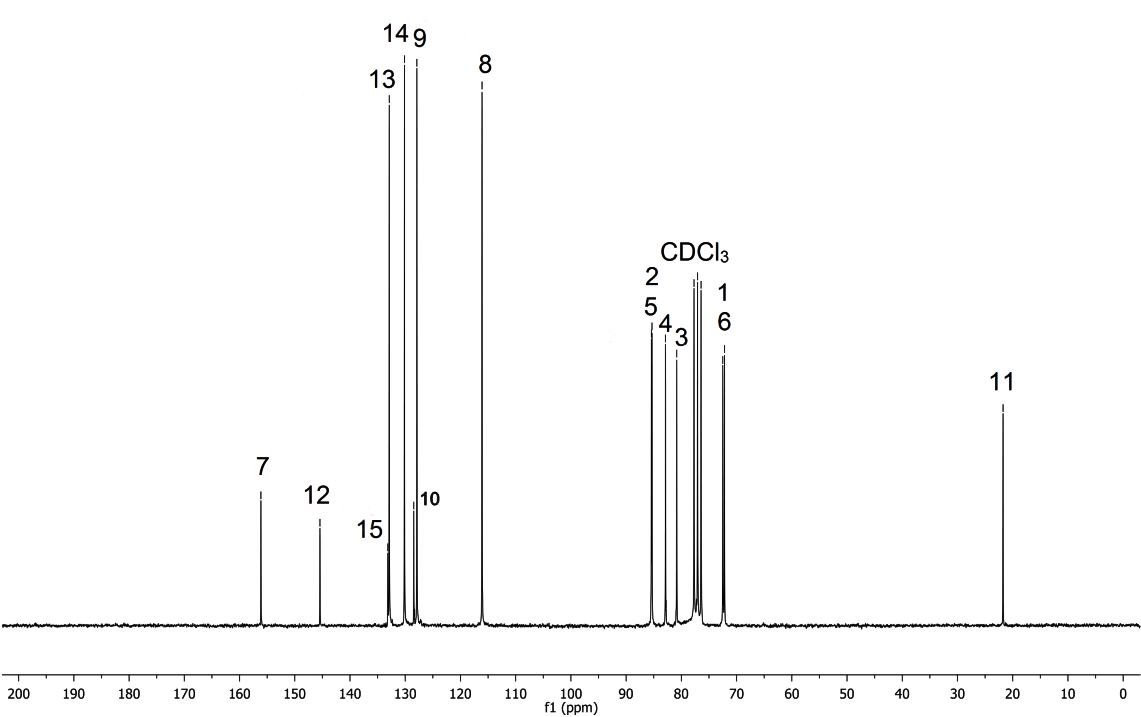


**Figure S6.** ^13^C NMR spectrum of the intermediate **THIO-ISDT** (**3b**) (in CDCl_3_)

**Figure S7.** TGA thermograms of **4a** (BPA-ISOH) in air and nitrogen.

**Figure S8.** TGA thermograms of **4b** (THIO-ISOH) in air and nitrogen.


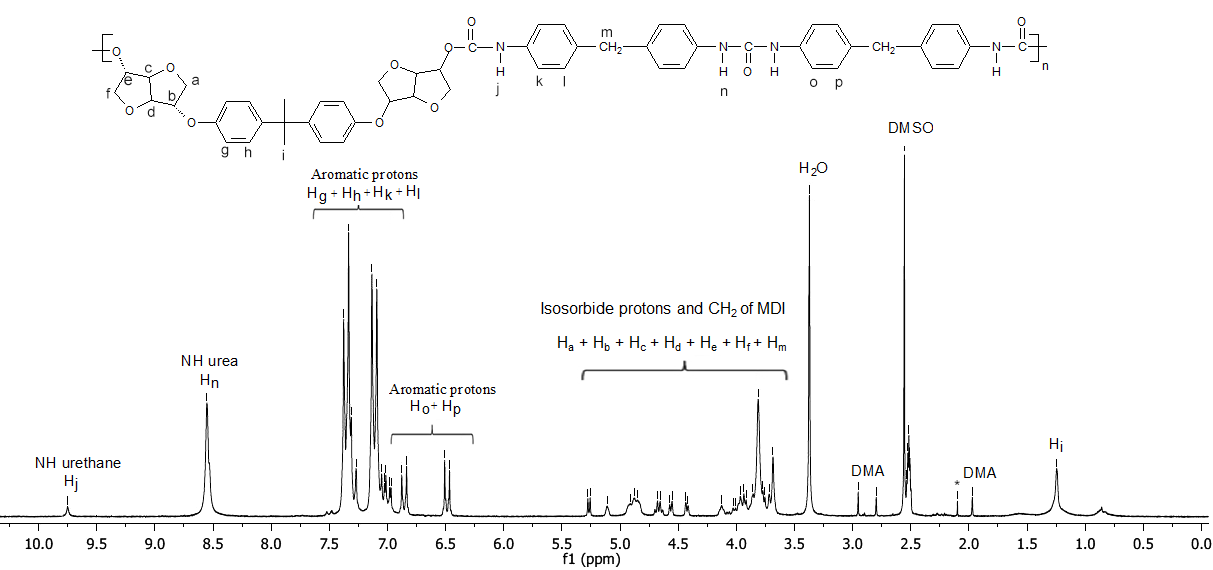


**Figure S9.** ^1^H NMR spectrum (DMSO-d_6_) of the polyurethane **PU(MDI)BPA**


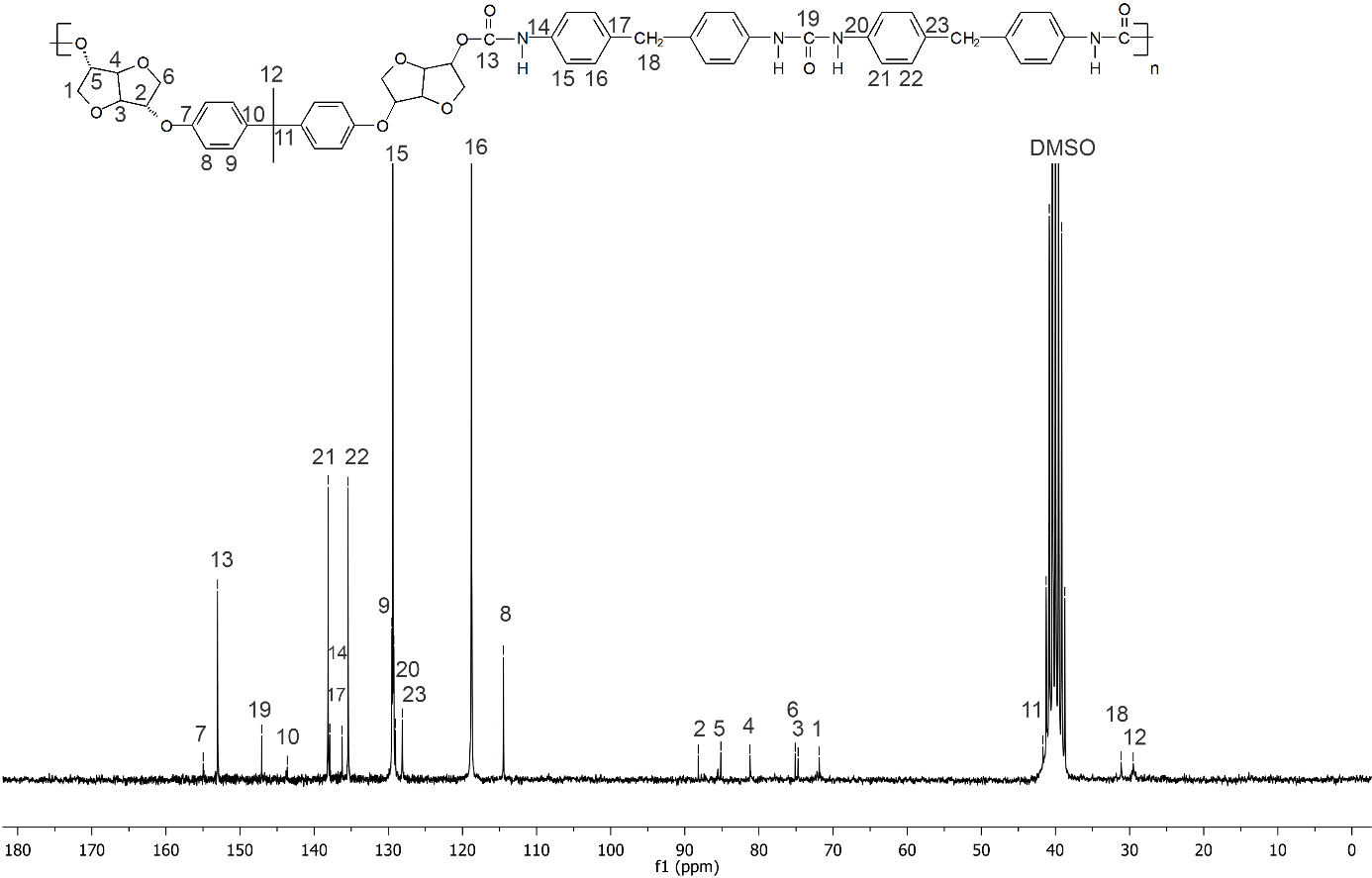


**Figure S10.** ^13^C NMR spectrum (DMSO-d_6_) of the polyurethane **PU(MDI)BPA**


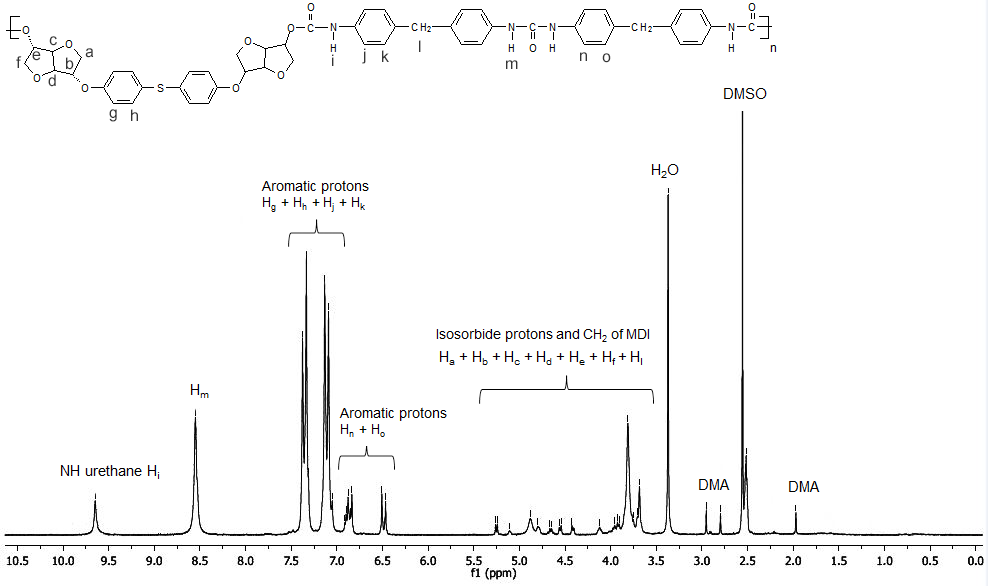


**Figure S11.** ^1^H NMR spectrum (DMSO-d_6_) of the polyurethane **PU(MDI)THIO**


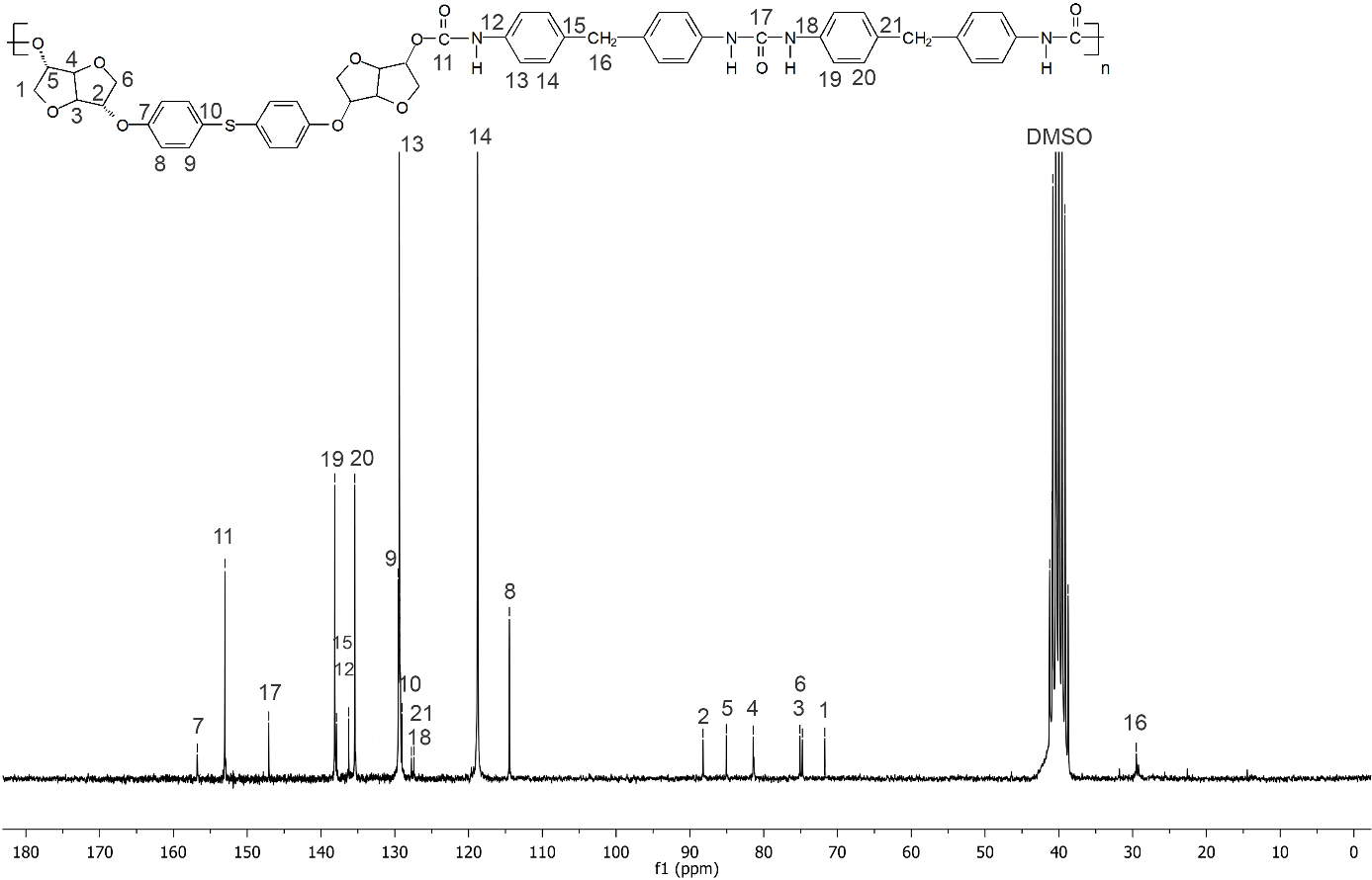


**Figure S12.** ^13^C NMR spectrum (DMSO-d_6_) of the polyurethane **PU(MDI)THIO**

**Figure S13.** DSC thermogram of **PU(HDI)BPA**
